# Optimising postoperative spine outcomes: an umbrella review of enhanced recovery after spinal surgery (ERASS) protocols

**DOI:** 10.1016/j.bja.2025.08.037

**Published:** 2025-09-05

**Authors:** Daniel Sescu, Devika Dahiya, Laura Scaramuzzo, Stipe Corluka, Sathish Muthu, Samuel K. Cho, Zorica Buser, Tim Sangwook Yoon, Andreas K. Demetriades

**Affiliations:** 1 The School of Medicine, Medical Sciences and Nutrition, University of Aberdeen, Aberdeen, UK; 2 Edinburgh Spinal Surgery Outcome Studies Group, Edinburgh, UK; 3 Department of Neurosciences, Aberdeen Royal Infirmary, Aberdeen, UK; 4 Spine Surgery Unit, Department of Aging, Orthopaedic and Rheumatological Sciences, Fondazione Policlinico Universitario Agostino Gemelli IRCCS, Rome, Italy; 5 AO Spine Knowledge Forum Degenerative, AO Network Clinical Research, AO Spine, AO Foundation, Davos, Switzerland; 6 Spinal Surgery Division, Department of Traumatology, University Hospital Centre Sestre Milosrdnice, Zagreb, Croatia; 7 Central Research Laboratory, Meenakshi Medical College Hospital and Research Institute, Meenakshi Academy of Higher Education and Research, Chennai, India; 8 Department of Orthopedic Surgery, Icahn School of Medicine at Mount Sinai, New York, NY, USA; 9 Department of Orthopedic Surgery, NYU Grossman School of Medicine, New York, NY, USA; 10 NY Orthopedics PC, Brooklyn, NY, USA; 11 Department of Orthopedic Surgery, Emory University, Atlanta, USA; 12 Department of Neurosurgery, Royal Infirmary Edinburgh, Edinburgh, UK; 13 Leiden University Medical Centre, University of Leiden, Leiden, The Netherlands

**Keywords:** enhanced recovery after surgery, ERAS, perioperative, protocol, spinal surgery, spine, surgery

## Abstract

**Background:**

Enhanced Recovery After Surgery (ERAS) protocols aim to improve recovery, reduce complications, and optimise surgical outcomes. Despite increasing use in spinal surgery, no standardised ERAS for spinal surgery (ERASS) exists and evidence synthesis is limited. This umbrella review consolidates findings from systematic reviews (SRs) and meta-analyses (MAs) to evaluate the clinical and economic impact of ERASS and identify research gaps.

**Methods:**

A systematic search of MEDLINE, Embase, Cochrane Database of Systematic Reviews, and Web of Science (1990–2024) identified SRs and MAs on ERASS. Data extraction followed Preferred Reporting Items for Systematic reviews and Meta-Analyses (PRISMA) and Preferred Reporting Items for Overviews of Reviews (PRIOR), with quality assessed using AMSTAR-2 and ROBIS. Overlapping primary studies were removed before recalculating pooled estimates using fixed or random-effects models based on heterogeneity. Primary outcomes included length of stay, postoperative complications, readmission rates, healthcare costs, pain scores, and opioid consumption.

**Results:**

Seventeen SRs and 55 MAs (319 primary studies; *n*=221 605 participants) were included. ERASS significantly reduced length of stay (−1.55 days; 95% confidence interval [CI] −1.83 to −1.27 days; *P*<0.01), postoperative complications (relative risk=0.61; 95% CI 0.52–0.72; *P*<0.01), opioid consumption (−7.26 mg morphine equivalents; 95% CI −10.82 to −3.70 mg; *P*<0.01), and healthcare costs (−$1029.41 per patient; 95% CI −$1630.17 to −$428.65; *P*<0.01). Readmission rates were not significantly impacted (relative risk=0.91; *P*=0.38). Pain scores showed a modest, non-significant reduction (−0.27; 95% CI −0.66 to 0.13; *P*=0.19). High heterogeneity was observed, reflecting protocol and design variation.

**Conclusions:**

ERASS protocols significantly improve surgical efficiency and safety. Standardised guidelines and future research addressing heterogeneity, under-represented ERASS elements, and long-term outcomes are needed.

**Systematic review protocol:**

PROSPERO (CRD42024578786).

**Editor’s key points**

• While Enhanced Recovery After Surgery (ERAS) protocols benefit other surgical specialties, evidence for spinal surgery (ERASS) remains fragmented and lacks standardised guidelines. ERASS has shown promise in reducing length of stay and complications, but impact on long-term outcomes and under-represented elements remains uncertain.
• This umbrella review synthesises evidence from 221 605 participants, confirming that ERASS significantly reduces hospital stay, complications, opioid use, and costs, although effects on readmissions and pain are less clear.
• The findings support ERASS adoption, highlighting gaps in long-term outcomes and understudied elements. Future work should prioritise multicentre RCTs to refine ERASS implementation.

Enhanced Recovery After Surgery (ERAS) protocols represent a significant advancement in perioperative care, designed to improve surgical outcomes through a comprehensive, evidence-based approach.^1^ Initially developed for colorectal surgery, ERAS protocols have been successfully adapted across various surgical specialties, including spinal surgery, to optimise patient recovery and reduce complications.^2^^,^^3^ These protocols integrate interventions across the entire clinical care pathway (i.e. preoperative, intraoperative, and postoperative phases), with key areas of focus such as patient education, pain management, fluid balance, and early mobilisation.^4^^,^^5^

Lower limb and lower back pain have a significant global impact.^6^^,^^7^ The outcomes of spinal surgery as a definitive treatment option are increasingly attracting attention worldwide.^8^^,^^9^ In the context of spinal surgery, where patients often undergo complex procedures with lengthy recovery periods, the implementation of ERAS protocols (primarily institution-specific and not yet widely validated) has shown promise in addressing the unique challenges of these surgeries.^10^^,^^11^ Traditionally, postoperative care in spinal surgery has involved extended bed rest and significant opioid use, both of which can hinder recovery and increase morbidity.^11^ To address these challenges, ERAS protocols advocate for strategies that promote quicker recovery and improved long-term outcomes, making them especially relevant to spinal surgery and functional recovery.^5^

The clinical significance of ERAS protocols lies in their ability to reduce the physiological stress of surgery, leading to better patient outcomes, shorter hospital stays, and lower healthcare costs.^12^ A multimodal approach to pain management within ERAS protocols helps reduce opioid dependence, minimising opioid-related side-effects and facilitating a quicker postoperative recovery.^13^ Early mobilisation and nutritional optimisation, integral components of ERAS, are crucial for accelerating functional recovery and minimising the risk of postoperative complications (i.e. infections and thromboembolic events).^14^

Systematic reviews (SRs) hold the highest level of evidence in the hierarchy and they are fundamental to evidence-based medicine and clinical guidelines. However, when multiple SRs on the same topic appear in the literature, their conclusions can conflict, creating uncertainty for decision-makers. Consequently, careful scrutiny of the quality of evidence from these reviews is essential. To address this challenge, an umbrella review (i.e. a systematic review of SRs) was introduced, serving as a valuable tool when several SRs have been published on a single topic.^15^ Umbrella reviews consolidate and critically evaluate existing SRs into one comprehensive synthesis, clarifying the overall evidence base, identifying consistencies and contradictions in the literature and gaps for future research.^16^

Interest in ERAS for spinal procedures has grown substantially, as reflected in recent literature.^17^ Despite the growing number of multiple SRs, meta-analyses (MAs) and institutional protocols published on ERAS in spinal surgery (ERASS), there is no universally accepted or standard ERAS pathway for spine surgery (except for lumbar fusion only).^18^ Currently, the ERAS Society’s website features 23 guidelines spanning various surgical specialties, yet only one pertains to spinal surgery (specifically lumbar fusion).^19^ Existing reviews focus on particular ERAS elements, noting inconsistent findings in certain outcomes (e.g. conflicting findings for postoperative pain, complications, hospital length of stay [LOS], and readmissions), leaving the effectiveness of ERAS in lumbar interbody fusion surgery still unclear.^17^ This fragmentation about which ERAS elements are effective creates confusion across the spectrum of spinal surgeries. These gaps in knowledge clarify the need for the present work.

Therefore, this umbrella review collates and analyses data on ERAS protocols in spinal surgery (ERASS) to provide a clear overview of the current evidence. In doing so, it highlights the efficacy of ERASS while also identifying areas where further research is needed. This might be the first step towards a standard summative guideline (integrating findings from multiple studies into a unified set of recommendations) for other spinal procedures and, more broadly, for spinal surgery as a whole, similar to what has already been achieved in other surgical specialties. As healthcare systems increasingly prioritise patient-centred care while facing mounting resource pressures, an integrated assessment of existing literature can help optimise the implementation of ERASS.

Ultimately, this umbrella review aims to provide a consolidated, evidence-based resource for clinicians and policymakers to inform and refine ERAS protocols in spinal surgery, enhancing patient outcomes and realising the full potential of ERAS in this complex field.

## Methods

This umbrella review was conducted in accordance with Preferred Reporting Items for Systematic reviews and Meta-Analyses (PRISMA)^20^ and Preferred Reporting Items for Overviews of Reviews (PRIOR)^16^ guidelines and is registered with PROSPERO (CRD42024578786).

### Search strategy and eligibility criteria

This study examined existing SRs and MAs of ERAS protocols in spinal surgery. Studies that were not SRs or MAs or that evaluated ERAS protocols in non-spinal surgeries were excluded. Only articles in English or translated into English were considered.

A systematic search was conducted in Ovid MEDLINE, Embase, Cochrane Database of Systematic Reviews, Centre for Reviews and Dissemination, and Web of Science from January 1, 1990 to May 26, 2024. A combination of keywords and subject headings related to ‘Enhanced Recovery After Surgery’, ‘ERAS’, ‘fast track’, ‘spinal surgery’, and ‘spine’ was used. Articles were screened independently (titles, abstracts) by two reviewers (DS, DD) for eligibility, with disagreements resolved by a third reviewer (AKD). Articles deemed potentially eligible underwent full-text review (reasons for exclusion were documented). Reference lists of included studies were reviewed for additional relevant SRs.

Consistent with our broad remit, ERASS studies were included across the entire spectrum of spinal procedures in both adult and paediatric populations. By explicitly embracing elective and emergency contexts, the most comprehensive overview of ERAS performance in real-world spine surgery is provided to date.

### Data extraction

Two reviewers (DS, DD) independently extracted data from each eligible SR and MA, including authorship, publication year, types (and number) of included studies, population characteristics, details of ERAS protocols, and reported outcomes. For MAs, we noted the effect estimates (mean difference [MD], relative risk [RR], odds ratio [OR]) with 95% confidence intervals (CIs). SRs that did not perform an MA were included in a narrative synthesis. Discrepancies in extraction were settled by discussion involving a third reviewer (AKD).

### Managing study overlap and discrepancies

We identified and recorded all primary studies within each SR and MA to detect overlapping data. Where multiple MAs provided pooled estimates of the same outcome using overlapping primary studies, the duplicates were removed before to any new pooled analysis. This prevented double-counting the same primary study in the final quantitative synthesis. If a primary study’s effect size or CI differed across reviews, we used the values reported in the original publication of the original publication.

### Outcomes of interest

The primary outcomes included: LOS, postoperative complications, readmission rates, postoperative patient-reported pain scores (within 24 h; recorded on a 0–10 scale [Visual Analogue or Numeric Rating Scale, i.e. VAS or NRS]), opioid consumption (morphine equivalent dosages within 24 h after surgery) and hospital costs (primary outcomes reported in our quantitative synthesis, i.e. pooled MAs). We also captured secondary outcomes when these were reported in SRs without MAs (reported in our narrative synthesis).

### Risk of bias, quality, and certainty assessments

Two reviewers (DS, DD) independently evaluated the methodological quality of included SRs using AMSTAR-2. ROBIS was then applied to determine the risk of bias in each SR. We relied on the risk-of-bias assessments reported in each SR for its included primary studies. We did not reassess each primary study unless there was a discrepancy or incomplete reporting. Lastly, the certainty of the evidence for each SR was assessed using the Grading of Recommendations, Assessment, Development and Evaluations (GRADE) (I [high], II [moderate], or III [low]) framework, determined during our data extraction.

### Statistical analysis

For the primary outcomes (reported in two or more MAs after removing overlapping primary studies), we conducted new pooled MAs (pooled MAs). Effect sizes (MD, OR, RR) were combined using either fixed-effects or random-effects models, guided by Cochran’s Q (*P*<0.10) and *I*^2^ thresholds (>50% indicated significant heterogeneity). Where appropriate, log-transformations of RRs were performed before back-transformation to stabilise variance, linearise effect sizes, and ensure symmetric CIs. All analyses were performed in STATA version 18 (Stata Corp. College Station, TX, USA). We quantified between-study variability using the *I*^2^ and *τ*^2^ statistics. We did not perform predefined subgroup analyses (e.g. specific ERASS interventions) because of inconsistencies in reporting ERASS components across reviews. We did not conduct formal sensitivity analyses (e.g. excluding low-quality SRs) owing to the limited number of high-quality SRs and MAs. Instead, heterogeneity was addressed via random-effects models and narrative contextualisation, with recommendations based on the current evidence on ERASS. Statistical significance was set at *P*<0.05.

### Reporting bias assessment

Publication bias and selective outcome reporting were assessed at the SR level using AMSTAR-2 criteria (e.g. Did the SR search multiple databases adequately? Did they investigate publication bias?). Potential reporting bias in the newly pooled analyses was qualitatively examined by comparing the overlap, effect sizes, and direction of findings across the included SRs. Funnel plots were not generated for the umbrella review because these are typically performed at the primary study level, which was beyond the scope of this study.

## Results

### Systematic review selection

A total of 110 records were identified, of which 28 were duplicates. After screening titles and abstracts of the remaining 82 records, 22 full-text articles were reviewed in detail. Ultimately, 17 SRs^10^^,^^21^, ^22^, ^23^, ^24^, ^25^, ^26^, ^27^, ^28^, ^29^, ^30^, ^31^, ^32^, ^33^, ^34^, ^35^, ^36^ and 55 associated MAs met inclusion criteria, encompassing 319 unique primary studies (after deduplication to avoid double-counting; *n*=221 605 participants). The PRISMA flow diagram detailing the study selection process is provided in Supplementary File 1. A list of the excluded studies (*n*=34 at title and abstract screening; *n*=5 at full-text assessment), along with reasons for exclusion, is provided in Supplementary File 2.

### Characteristics of included systematic reviews

The 17 SRs^10^^,^^21^, ^22^, ^23^, ^24^, ^25^, ^26^, ^27^, ^28^, ^29^, ^30^, ^31^, ^32^, ^33^, ^34^, ^35^, ^36^ included in this umbrella review covered various types of spinal surgery (e.g. lumbar fusion, cervical decompression, thoracolumbar instrumentation) and were published between 2017 and 2024 (16 SRs in 2019; rate of publication: approximately three SRs per year). Table 1 summarises each SR (citation, population, intervention, comparator, outcomes, number and type of primary studies, funding, conflicts, quality [AMSTAR-2], risk of bias [ROBIS], GRADE of evidence, funding, and conflicts).

**Table 1:** Characteristics of systematic reviews included in the umbrella review of Enhanced Recovery After Surgery (ERAS) protocols in spinal surgery. ERAS, enhanced recovery after surgery; ESPB, erector spinae plane block; PCA, patient-controlled analgesia; PONV, postoperative nausea and vomiting; RCT, randomised controlled trial; RoB, risk of bias; SSI, surgical site infection.

Authors, year	Title	Population	Intervention	Comparator	Outcome	Types of studies	AMSTAR 2	ROBIS	Grade of evidence	Funding + conflicts
Magableh and colleagues, 2024^36^	Transforming outcomes of spine surgery—exploring the power of enhanced recovery after surgery protocol: a systematic review and meta-analyses of 15 198 patients	ERAS *n*=7748 Control *n*=7450 Adult population (≥18 yr), region (cervical, thoracic, lumbar, or mixed), and type of spine surgery (laminectomy, fusion, or mixed)	ERAS protocol (≥3 standardised interventions in the protocol)	Traditional perioperative care regimen	Operative time estimated blood loss Opioid use Pain scores Time to mobilisation Length of stay Complication rate Readmission rate Reoperation rate Oswestry disability index Healthcare costs	59 Studies 4 RCTs 4 Prospective cohort 51 Retrospective cohort	Low quality	Low RoB	Grade III	No funding received No conflicts of interest
Qin and colleagues, 2024^35^	A meta-analysis of the implementation of enhanced recovery after surgery pathways in anterior cervical spine surgery for degenerative cervical spine diseases	ERAS *n*=1306 Control *n*=1430 All patients undergoing anterior cervical spine surgery for the treatment of degenerative cervical spine disease. No age or gender restrictions	ERAS protocol Pre-, intra- and postoperative phases	Traditional perioperative care regimen	Length of stay Complication rate Readmission rate Reoperation rate Healthcare costs Patient satisfaction	10 Studies 5 RCTs 2 Prospective cohort 3 Retrospective cohort	Low quality	Low RoB	Grade I	Numerous sources of funding reported No conflicts of interest
Wilson and colleagues, 2024^34^	Erector spinae plane block on postoperative pain and opioid consumption after lumbar spine surgery: a systematic review and meta-analysis of randomised controlled trials	*n*=1327 Patients undergoing lumbar spine surgeries (lumbar fusion, elective decompressive, laminoplasty, herniated disc microdiscectomy lumbar spinal surgery)	ESPB	Standard analgesia	Opioid use Time to first rescue analgesia Pain scores PONV Patient satisfaction	22 RCTs	High quality	Low RoB	Grade I	No funding received No conflicts of interest
Contartese and colleagues, 2023^33^	Fast-track protocols for patients undergoing spine surgery: a systematic review	Fast-track *n*=11 385 Control *n*=6040 Patients undergoing elective spine surgery (including minor, major, and complex surgeries)	Fast-track protocol	Traditional perioperative care regimen	Estimated blood loss Length of stay Complication rate Opioid use Pain scores	57 Studies 8 Prospective cohort 49 Retrospective cohort	Low quality	Low RoB	Grade III	Funded by grants from IRCCS Instituto Ortopedico Rizzoli and by 5X1000 2018 project (PRWEB: 2020/730420) No conflicts of interest
Bae and colleagues, 2022^32^	Efficacy of perioperative pharmacological and regional pain interventions in adult spine surgery: a network meta-analysis and systematic review of randomised controlled trials	*n*=6284 Adults (>18 yr) undergoing spine surgery under general anaesthesia, irrespective of the pathologies, location, number of levels, and complexity of surgery	ERAS elements	Traditional perioperative care regimen	Pain scores Opioid use	86 RCTs	Moderate quality	Low RoB	Grade I	Funding not reported No conflicts of interest
Salamanna and colleagues, 2022^31^	Key components, current practice and clinical outcomes of ERAS programs in patients undergoing orthopaedic surgery: a systematic review	*n*=not reported Patients undergoing orthopaedic surgery (spinal surgery included)	ERAS protocol Pre-, intra- and postoperative phases	Traditional perioperative care regimen	Operative time Length of stay Opioid use Complication rate Readmission rate Healthcare costs	25 Spinal studies	Low quality	Low RoB	Grade III	Funded by grants from IRCCS Instituto Ortopedico Rizzoli and by 5X1000 2018 project (PRWEB: 2020/730420) No conflicts of interest
Zaed and colleagues, 2022^30^	Current state of benefits of Enhanced Recovery After Surgery (ERAS) in spinal surgeries: a systematic review of the literature	*n*=2585 Patients undergoing cervical, lumbar spine surgeries and correction of scoliosis and spinal deformity	ERAS protocol	Control group	Length of stay Pain scores Readmission rate Reoperation rate Patient satisfaction	21 Studies 1 RCT 8 Prospective cohort 12 Retrospective cohort	Low quality	Low RoB	Grade II	Funding not reported No conflicts of interest
Koucheki and colleagues, 2021^29^	Comparison of interventions and outcomes of enhanced recovery after surgery: a systematic review and meta-analysis of 2456 adolescent idiopathic scoliosis cases	*n*=2456 Patients undergoing surgical management for adolescent idiopathic scoliosis	ERAS protocol	Traditional perioperative care regimen	Operative time Estimated blood loss Opioid use PCA discontinuation Pain scores Time to mobilisation Length of stay Complication rate Readmission rate	14 Studies 1 Prospective cohort 13 Retrospective cohort	Moderate quality	Low RoB	Grade III	Funding not reported No conflicts of interest
Licina and colleagues, 2021^28^	Pathway for enhanced recovery after spinal surgery: a systematic review of evidence for use of individual components	*n*=not reported Adult and paediatric patients undergoing spinal surgical procedure on any spinal anatomical site	ERAS protocol	Traditional perioperative care regimen	Pain scores Length of stay Postoperative nausea an vomiting complications	664 Studies Study type breakdown not reported—mixed RCTs, prospective and retrospective cohort studies	Low quality	Low RoB	Grade II	No funding received No conflicts of interest
Liang and colleagues, 2021^27^	Erector spinae plane block for spinal surgery: a systematic review and meta-analysis	*n*=696 Patients undergoing spinal surgery	ESPB	Standard analgesia	Opioid use Pain scores Time to rescue analgesia Postoperative nausea and vomiting Urinary retention Itching Time to mobilisation Length of stay	12 RCTs	Moderate quality	Low RoB	Grade I	No funding received No conflicts of interest
Pennington and colleagues, 2021^26^	Systematic review and meta-analysis of the clinical utility of enhanced recovery after surgery pathways in adult spine surgery	*n*=8194 Adult patients undergoing any type of elective spine surgery	ERAS protocol	Traditional perioperative care regimen	Length of stay Complication rate Wound infection Readmission rate Reoperation rate Healthcare costs	34 Studies in qualitative analysis 20 Studies in quantitative analysis Study type breakdown not reported—mixed RCTs, prospective and retrospective cohort studies	Low quality	Low RoB	Grade III	Funding not reported Potential conflicts disclosed for 2 authors with various industry partners
Tan and colleagues, 2020^25^	Prophylactic postoperative measures to minimise surgical site infections in spine surgery: systematic review and evidence summary	RCTs: median 155 (range: 30–326) Cohort studies: median 265.5 (range: 42–10 225)	ERAS protocol	Traditional perioperative care regimen	Overall rate of SSI (superficial and deep SSI)	9 RCTs 32 Cohort studies	Moderate quality	Low RoB	Grade II	No funding received No conflicts of interest
Tong and colleagues, 2020^24^	Enhanced recovery after surgery trends in adult spine surgery: a systematic review	*n*=13 655 Adult patients undergoing any type of elective spine surgery	ERAS protocol	Traditional perioperative care regimen	Length of stay Healthcare costs Opioid use	22 Studies 12 Controlled before-and-after studies 8 Prospective cohort 2 Retrospective cohort	Low quality	Low RoB	Grade III	No funding received No conflicts of interest
Burgess and colleagues, 2019^23^	The effect of preoperative education on psychological, clinical and economic outcomes in elective spinal surgery: a systematic review	Adults (≥18 yr) receiving spine surgery	ERAS protocol	Traditional perioperative care regimen	Pain scores Psychological outcomes Quality of fife Complication rate Healthcare costs	7 RCTs	Low quality	Low RoB	Grade I	No funding received Potential conflicts disclosed for one author with the Enhanced Recovery after Surgery Society (Company No. 10932208)
Dietz and colleagues, 2019^22^	Enhanced Recovery After Surgery (ERAS) for spine surgery: a systematic review	*n*=not reported Operative adult patients (≥18 yr) undergoing spinal surgery	ERAS protocol	Traditional perioperative care regimen	Length of stay Pain scores Opioid use Complication rate Reoperation rate Healthcare costs	19 Studies Study type breakdown not reported: mixed RCTs, prospective and retrospective cohort studies 14 Comparative analysis studies 5 Non-comparative studies	Moderate quality	Low RoB	Grade II	Funding not reported No conflicts of interest
Elsarrag and colleagues, 2019^10^	Enhanced recovery after spine surgery: a systematic review	*n*=not reported Operative adult (≥18 yr) patients undergoing spinal surgery	ERAS protocol	Traditional perioperative care regimen	Opioid use Time to Mobilisation Complications Length of stay	20 Studies	Low quality	Low RoB	Grade III	Funding not reported No conflicts of interest
Peng and colleagues, 2017^21^	Gabapentin can decrease acute pain and morphine consumption in spinal surgery patients	Gabapentin *n*=383 Control *n*=198 Patients undergoing spine surgery	Gabapentin	Traditional analgesia	Pain scores Opioid use Complications PONV	7 RCTs	Moderate quality	Low RoB	Grade I	No funding received No conflicts of interest

Ten SRs were assessed as low quality by AMSTAR-2, five as moderate quality, and two as high quality. Most papers classified as low quality by AMSTAR-2 lacked protocol registration, failed to list excluded studies, did not address publication bias, and omitted reporting of funding sources. All SRs demonstrated low overall risk of bias by ROBIS criteria, with minimal concerns regarding specifications for study eligibility, methods used to identify studies, collect data, appraise the studies and synthesise findings. The certainty of evidence and recommendations (GRADE) was rated as I (high) for six SRs (five of which were entirely comprised of RCTs), II (moderate) for three SRs (mostly including prospective cohort studies and before-and-after comparative analyses), and III (low) for eight SRs (mostly reporting retrospective cohort studies) (Table 1). Seven SRs conducted at least two MAs each, while the remaining 10 provided narrative syntheses only.

### Enhanced recovery after spinal surgery elements from included studies

**Fig 1:**
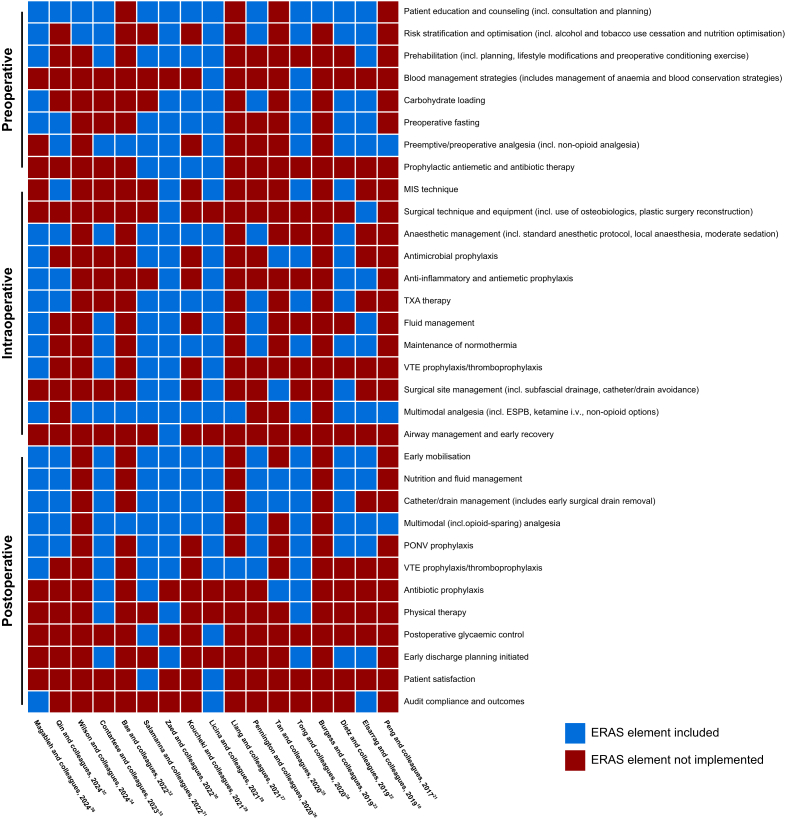
Implementation of Enhanced Recovery After Surgery (ERAS) elements across perioperative phases in spine surgery. Heatmap illustrating ERAS elements reported in the included studies (*n*=17) from the umbrella review. ESPB, erector spinae plane block; MIS, minimally invasive surgery; PONV, postoperative nausea and vomiting; TXA, tranexamic acid; VTE, venous thromboembolism.

Intervention is any ERAS element integrated in an included study. Often, multiple individual elements were combined into an overarching ERASS protocol specific to the study design and compared with a traditional perioperative care regimen. Figure 1 summarises the individual ERASS elements incorporated into each respective SR. These elements are broken down into preoperative, intraoperative, and postoperative interventions.

### Pooled meta-analyses

Within the included SRs, 55 MAs (from seven SRs) reported summary effect sizes for one or more of the six prespecified primary outcomes and secondary outcomes. After deduplicating overlapping primary studies, the total number of unique primary studies was 319 (*n*=221 605 participants) (references for the primary studies are found in Supplementary File 3). Table 2 shows MAs from primary studies, the number of cases and controls, the summary effect size, the largest study by the number of participants with their corresponding effect size and CI, heterogeneity measured (*I*^2^), and corresponding *P*-values.

**Table 2:** Meta-analysis results from included systematic reviews incorporating Enhanced Recovery After Surgery (ERAS) protocols for reported outcomes. CI, confidence interval; EBL, estimated blood loss; *I*^2^, heterogeneity statistic; ICU, intensive care unit; MD, mean difference; NR, not reported; ODI, Oswestry disability index; OR, odds ratio; PCA, patient-controlled analgesia; PI, prediction interval; POD, postoperative day; RR, risk ratio; se, standard error; VAS, visual analogue scale. Patient-reported pain scores.

Meta-analysis	Description	No. of primary studies	No. of cases/controls (total)	Random-effects summary effect size (95% CI)	95% PI	*P* random	*P* fixed	Largest study by no. of participants		*I*^2^ (%)	*P* for *I*^2^
								Effect size (95% CI)	se		
Length of stay											
Magableh and colleagues, 2024^36^		54	6101/6031	MD: −1.41 (−1.76 to −1.05)	−3.96 to 1.15	<0.01	NR	−1.90 (−2.29 to −1.51)	0.20	98	NR
Qin and colleagues, 2024^35^		10	1306/1430	MD: −2.16 (−2.57 to −1.75)	NR	<0.01	NR	−1.78 (−1.87 to −1.69)	0.05	89	<0.01
Koucheki and colleagues, 2021^29^		14	1375/1081	MD: −1.60 (−1.78 to −1.41)	NR	<0.01	NR	−1.30 (−1.56 to −1.04)	0.09	93	<0.01
Pennington and colleagues, 2021^26^		20	3159/3524	MD: −1.22 (−1.98 to −0.47)	NR	<0.01	NR	−1.37 (−1.55 to −1.19)	0.64	94	<0.01
Postoperative complication rate											
Magableh and colleagues, 2024^36^		49	5634/5583	RR: 0.64 (0.53–0.77)	NR	<0.01	NR	0.74 (0.58–0.95)	0.09	52	<0.01
Qin and colleagues, 2024^35^		6	982/1121	OR: 0.68 (0.51–0.91)	NR	NR	<0.01	1.39 (0.92–2.11)	0.30	88	<0.01
Koucheki and colleagues, 2021^29^		5	591/488	OR: 0.70 (0.44–1.10)	NR	NR	0.12	0.40 (0.15–1.08)	0.51	59	0.04
Pennington and colleagues, 2021^26^		5	2753/3753	OR: 0.81 (0.57–1.15)	NR	0.10	NR	0.98 (0.79–1.22)	0.11	48	0.10
Readmission rate											
Magableh and colleagues, 2024^36^		25	3533/3805	RR: 0.80 (0.60–1.07)	NR	0.13	NR	1.98 (1.24–3.18)	0.50	26	0.13
Qin and colleagues, 2024^35^		4	1077/1217	OR: 0.63 (0.30–1.35)	NR	NR	0.24	0.68 (0.30–1.56)	0.32	0	0.71
Koucheki and colleagues, 2021^29^		7	603/747	RR: 1.30 (0.61–2.78)	NR	0.51	NR	1.20 (0.32–4.55)	0.68	0	0.63
Pennington and colleagues, 2020^26^		5	2753/3753	OR: 0.87 (0.67–1.14)	NR	0.33	NR	0.90 (0.65–1.24)	0.15	0	0.90
Cost											
Magableh and colleagues, 2024^36^		13	1485/1535	MD: −1140.26 (−1843.99 to −436.53)	−3755.01 to 1474.49	<0.01	NR	−2865.00 (−3112.83 to −2617.17)	126.43	98	NR
Qin and colleagues, 2024^35^		5	216/219	MD: −0.81 (−1.08 to −0.53)	NR	NR	<0.01	Not estimable	NR	23	0.28
Patient-reported pain scores											
Magableh and colleagues, 2024^36^	Overall cumulative POD0–2 at rest	17	1617/1803	MD: 0.56 (0.21–0.91)	−0.98 to 2.09	<0.01	NR	0.67 (0.54–0.80)	0.07	95	NR
Wilson and colleagues, 2024^34^	Overall cumulative POD0–2 during activity	19	(2847)	MD: −1.03 (−1.19 to −0.87)	NR	NR	NR	NR	NR	98	<0.01
Wilson and colleagues, 2024^34^	POD0 at rest	11	1265/1265	MD: −1.16 (−0.83 to −0.34)	NR	<0.01	NR	0.00 (−0.01 to 0.01)	<0.01	99	<0.01
Liang and colleagues, 2021^27^	POD1 at rest	3	90/90	MD: −2.41 (−3.32 to −1.50)	NR	<0.01	NR	−1.70 (−1.96 to −1.44)	0.13	91	<0.01
Liang and colleagues, 2021^27^	POD2 at rest	6	180/180	MD: −0.54 (−1.06 to −0.01)	NR	0.04	NR	−1.00 (−1.20 to −0.80)	0.10	93	<0.01
Liang and colleagues, 2021^27^	POD0 at movement	4	120/120	MD: −0.21 (−0.47 to 0.06)	NR	0.12	NR	0.00 (−0.25 to 0.25)	0.13	51	0.11
Liang and colleagues, 2021^27^	POD1 at movement	3	90/90	MD: −2.67 (−2.96 to −2.38)	NR	<0.01	NR	−2.50 (−2.91 to −2.09)	0.21	0	0.38
Liang and colleagues, 2021^27^	POD2 at movement	7	208/208	MD: −1.01 (−1.61 to −0.42)	NR	<0.01	NR	−1.50 (−1.66 to −1.34)	0.08	93	<0.01
Liang and colleagues, 2021^27^	POD0 (VAS)	4	120/120	MD: −0.46 (−0.88 to −0.03)	NR	0.04	NR	−0.60 (−0.91 to −0.29)	0.16	72	0.01
Koucheki and colleagues, 2021^29^	POD1 (VAS)	4	204/298	MD: −1.12 (−1.29 to −0.94)	NR	NR	<0.01	−1.10 (−1.38 to −0.82)	0.14	0	0.48
Koucheki and colleagues, 2021^29^	POD2 (VAS)	4	250/336	MD: −0.88 (−1.06 to 0.70)	NR	NR	<0.01	−1.20 (−1.81 to −0.59)	0.31	90	<0.01
Koucheki and colleagues, 2021^29^	POD1 (VAS)	4	250/336	MD: −0.85 (−1.03 to −0.68)	NR	NR	<0.01	−0.50 (−0.73 to −0.27)	0.12	94	<0.01
Peng and colleagues, 2017^21^		4	293/108	MD: −1.04 (−1.29 to −0.79)	NR	<0.01	NR	−0.99 (−1.40 to −0.59)	0.21	83	<0.01
Opioid consumption (mg)											
Magableh and colleagues, 2024^36^	Total morphine equivalent dose (mg)	10	727/963	MD: −164.36 (−252.35 to −76.37)	−475.32 to 146.60	<0.01	NR	−217.30 (−336.36 to −98.24)	60.77	96	NR
Wilson and colleagues, 2024^34^	Opioid consumption (mg)	19	(1089)	MD: −6.25 (−8.33 to −4.17)	NR	NR	NR	NR	NR	NR	NR
Liang and colleagues, 2021^27^	Cumulative intraoperative i.v. morphine equivalent consumption (mg)	7	NR	MD: −9.63 (−15.40 to −3.86)	NR	<0.01	NR	NR	NR	93	<0.01
Peng and colleagues, 2017^21^	Opioid consumption (mg)	6	353/168	MD: −2.04 (−2.71 to −1.37)	NR	<0.01	NR	−5.8 (−6.83 to −4.76)	0.53	93	<0.01
First time of ambulation (days)											
Liang and colleagues, 2021^27^		3	NR	RR: −0.93 (−1.97 to 0.07)	NR	0.07	NR	NR	NR	98	<0.01
Pruritus											
Liang and colleagues, 2021^27^		5	NR	RR: 0.58 (0.25–1.35)	NR	0.21	NR	NR	NR	31	0.22
Peng and colleagues, 2017^21^		4	123/123	RR: 0.38 (0.22–0.66)	NR	<0.01	NR	0.41 (0.19–0.88)	0.18	0	0.96
PONV											
Wilson and colleagues, 2024^34^		6	198/198	RR: 0.29 (0.10–0.79)	NR	0.02	NR	0.07 (0.01–0.58)	0.14	43	0.12
Liang and colleagues, 2021^27^		9	NR	RR: 0.54 (0.36–0.83)	NR	<0.01	NR	NR	NR	40	0.10
Peng and colleagues, 2017^21^		6	208/173	RR: 0.46 (0.27–0.78)	NR	<0.01	NR	0.27 (0.10–0.73)	0.16	0	0.82
Urinary retention											
Liang and colleagues, 2021^27^		2	NR	RR: 0.50 (0.10–2.60)	NR	0.41	NR	NR	NR	0	1.00
Peng and colleagues, 2017^21^		4	123/123	RR: 0.57 (0.34–0.98)	NR	0.04	NR	0.56 (0.21– 1.30)	0.28	28.5	0.24
Urinary catheter discontinuation (days)											
Koucheki and colleagues, 2021^29^		4	333/289	MD: −0.51 (−0.59 to −0.43)	NR	NR	<0.01	−0.50 (−0.62 to −0.38)	0.06	0	0.79
Rescue analgesia											
Wilson and colleagues, 2024^34^		10	271/269	RR: 0.33 (0.13–0.83)	NR	0.02	NR	0.21 (0.10–0.41)	0.08	95	<0.01
Liang and colleagues, 2021^27^		7	NR	RR: 0.39 (0.19–0.80)	NR	0.01	NR	NR	NR	90	<0.01
Time to first rescue analgesia (h)											
Liang and colleagues, 2021^27^		5	NR	MD: −6.15 (−10.12 to −2.19)	NR	0.01	NR	NR	NR	100	<0.01
Operative time (min)											
Magableh and colleagues, 2024^36^		40	4099/4085	MD: −10.29 (−17.86 to −2.71)	−56.21 to 35.64	< 0.01	NR	−6.10 (−9.61 to −2.59)	1.79	92	NR
Koucheki and colleagues, 2021^29^		8	680/496	MD: −35.56 (−68.36 to −2.76)	NR	0.03	NR	−92 (−105.88 to −78.12)	7.08	99	<0.01
EBL (ml)											
Magableh and colleagues, 2024^36^		33	2856/3058	MD: −49.77 (−70.19 to −29.35)	−156.19 to 56.65	<0.01	NR	−29.80 (−32.81 to −26.79)	1.54	92	NR
Koucheki and colleagues, 2021^29^		6	664/436	MD: −112.92 (−122.16 to −102.42)	NR	NR	<0.01	−427 (−536.29 to −317.71)	55.76	99	<0.01
Time to mobilisation											
Magableh and colleagues, 2024^36^	(days)	10	667/706	MD: −0.92 (−1.33 to −0.50)	−2.47 to 0.64	<0.01	NR	−0.95 (−1.38 to −0.52)	0.22	96	NR
Koucheki and colleagues, 2021^29^	(h)	3	202/193	MD: −29.63 (−48.04 to −11.22)	NR	0.002	NR	−26.40 (−30.57 to −22.23)	2.13	97	<0.01
Change in ODI											
Magableh and colleagues, 2024^36^		10	1029/1149	MD: 2.06 (−0.01 to 4.13)	−5.34 to 9.45	0.05	NR	3.8 (2.63–4.97)	0.60	91	NR
Postoperative dysphagia											
Qin and colleagues, 2024^35^		2	272/275	OR: 0.60 (0.27–1.31)	NR	NR	0.20	1.68 (0.40–7.14)	1.72	66	0.09
Patient satisfaction comparison											
Qin and colleagues, 2024^35^		4	168/170	OR: 3.13 (1.97–4.98)	NR	NR	<0.01	4.19 (1.90–9.25)	1.88	5	0.37
Time to PCA discontinuation (days)											
Koucheki and colleagues, 2021^29^		5	369/325	MD: −0.53 (−0.58 to −0.47)	NR	NR	<0.01	−0.43 (−0.58 to −0.28)	0.0765	97	<0.01
Wound infection											
Pennington and colleagues, 2021^26^		3	2986/2642	OR: 0.78 (0.46–1.34)	NR	0.37	NR	0.91 (0.57–1.45)	0.238	55	0.11
ICU readmission											
Pennington and colleagues, 2021^26^		2	468/182	OR: 1.10 (0.72–1.68)	NR	0.67	NR	1.26 (0.80–1.98)	0.231	12	0.29

In addition, we conducted new pooled MAs for the six primary outcomes after deduplication. These outcomes were selected based on the availability of data from at two or more MAs with different contributing primary studies.

### Length of stay

**Fig 2:**
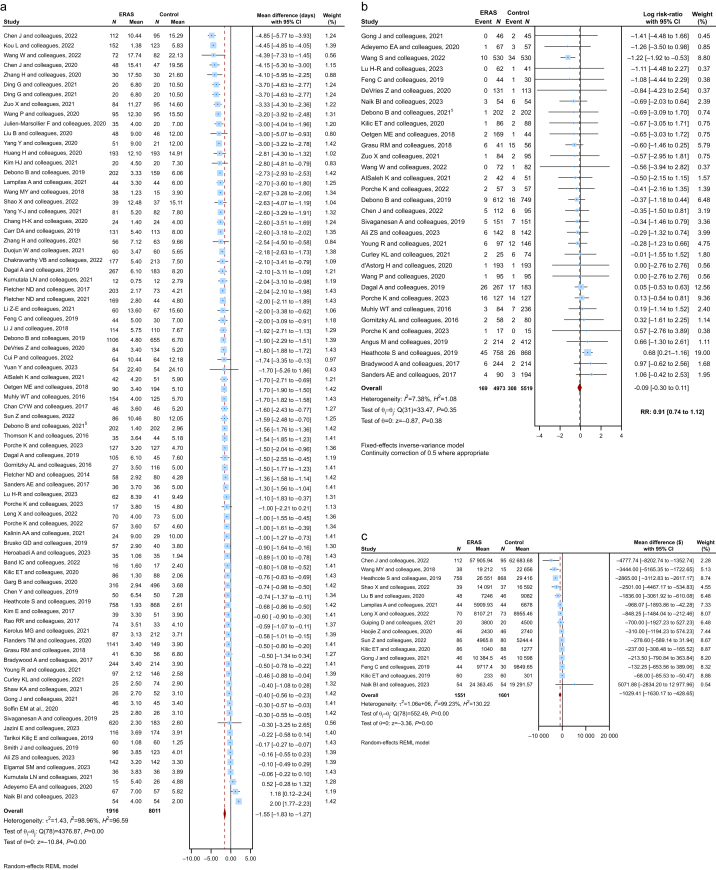
Length of hospital stay, readmission rate, and cost. (a) Length of hospital stay: Forest plot displaying the results of the quantitative meta-analysis (n = 79 primary studies). A random-effects model was used. ERAS protocols were associated with a reduced number of hospital days. (b) Readmission rate: Forest plot showing the results of the quantitative meta-analysis (n = 32 primary studies). Log risk ratios (log-RR) were analysed and presented as risk ratios (RR). A fixed-effects model was applied. ERAS protocols were associated with reduced risk of readmission. C. Cost: Forest plot summarising the results of the quantitative meta-analysis (n = 15 primary studies). A random-effects model was used. ERAS protocols were associated with reduced total cost per patient (in US dollars). Abbreviations: ERAS, enhanced recovery after surgery; MD, mean difference; SD, standard deviation; CI, confidence interval; REML, restricted maximum likelihood; RR, risk ratio. See Supplementary File 3 for the full list of studies included in the meta-analyses.

A total of 79 primary studies (*n*=17 627) reported on LOS. Using a random-effects model, ERASS protocols were associated with a significant mean reduction of 1.55 days (95% CI −1.83 to −1.27; *P*<0.01). High heterogeneity was observed (*I*^2^=99.0%; *τ*^2^=1.43), indicating considerable variability in ERASS implementation and patient populations (Fig. 2a). Despite this heterogeneity, nearly all contributing studies favoured ERASS over traditional care.

### Postoperative complications

**Fig 3:**
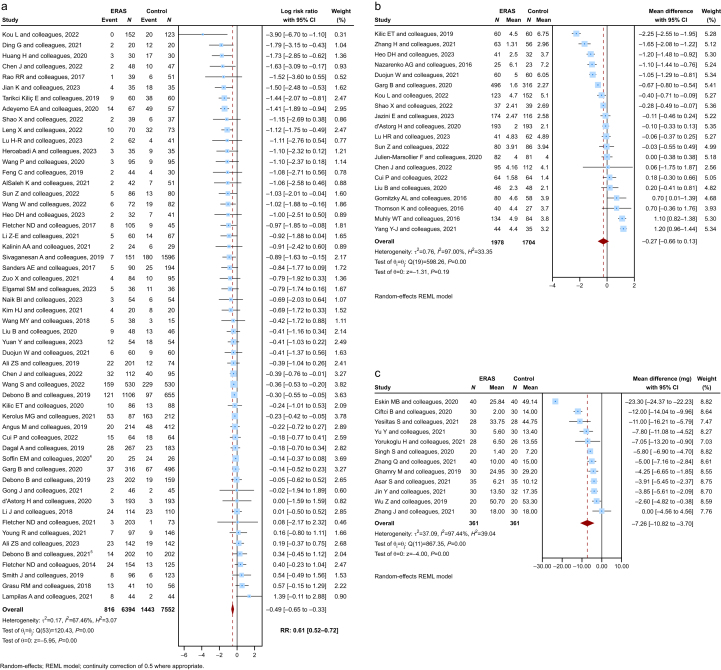
Postoperative complication rate, change in patient reported pain scores, and opiod consumption. (a) Postoperative complication rate: Forest plot presenting the results of the quantitative meta-analysis (n = 54 primary studies). Log risk ratios (log-RR) were analysed and reported as risk ratios (RR). A random-effects model was used. ERAS protocols were associated with a reduced rate of postoperative complications following spine surgery. (b) Change in patient-reported pain scores: Forest plot displaying the results of the quantitative meta-analysis (n = 20 primary studies). A random-effects model was used. ERAS protocols were associated with lower postoperative pain scores, measured using the visual analogue scale (VAS) or numerical rating scale (NRS). C. Opioid consumption: Forest plot showing the results of the quantitative meta-analysis (n = 12 primary studies). A random-effects model was used. ERAS protocols were associated with lower postoperative morphine consumption (mg). Abbreviations: ERAS, enhanced recovery after surgery; REML, restricted maximum likelihood; RR, risk ratio; MD, mean difference; SD, standard deviation; CI, confidence interval; VAS, visual analogue scale; NRS, numerical rating scale. See Supplementary File 3 for the full list of studies included in the meta-analyses.

Postoperative complications were evaluated in 54 primary studies (*n*=13 946). ERASS significantly reduced complication rates with a pooled log-RR of −0.49 (95% CI −0.65 to −0.33), back-transformed to an RR of 0.61 (95% CI 0.52–0.72; *P*<0.01). Moderate heterogeneity was noted (*I*^2^=67.5%; *τ*^2^=0.17), reflecting differences in complication definitions and types of spinal procedures among studies (Fig. 3a).

### Readmission rates

Thirty-two primary studies (*n*=10 492) reported on hospital readmissions. Given low heterogeneity (*I*^2^=7.4%), a fixed-effects model was applied. The resulting pooled RR of 0.91 (95% CI 0.74–1.12; *P*=0.38) indicated a non-significant 9% relative reduction in readmissions with ERASS (Fig. 2b). No *τ*^2^ was computed because of the fixed-effects approach.

### Hospital costs

Hospital cost data were extracted from 15 primary studies (*n*=3152). Random-effects pooling demonstrated a significant mean cost reduction of US $1029.41 per patient (95% CI US $−1630.17 to US $−428.65; *P*<0.01) favouring ERASS (Fig. 2c). However, heterogeneity was substantial (*I*^2^=99.2%; *τ*^2^=1.06×10^6^), highlighting the diversity in cost accounting practices, healthcare settings, and ERASS protocols.

### Patient-reported pain scores

Pain scores within the first 24 postoperative hours (VAS or NRS were reported by 20 studies [*n*=3682]). A random-effects model showed a modest non-significant MD of −0.27 (95% CI −0.66 to 0.13; *P*=0.19) favouring ERASS (Fig. 3b). Heterogeneity was high (*I*^2^=97.0%; *τ*^2^=0.76), likely reflecting variations in analgesic regimens and pain assessment tools.

### Morphine consumption

Twelve studies (*n*=722) reported morphine use in the first 24 h after surgery. ERASS protocols yielded a significant −7.26 mg reduction in morphine consumption (95% CI −10.82 to −3.70; *P*<0.01) under a random-effects model (Fig. 3c). The analysis showed high heterogeneity (*I*^2^=97.4%; *τ*^2^=37.09), which may be attributed to differences in multimodal analgesia and opioid-sparing strategies across centres.

### Narrative synthesis

Our narrative synthesis, summarised in Table 3, draws on findings from 10 SRs spanning GRADE I, II, and III evidence. Collectively, these SRs evaluate a range of spine-related procedures and ERASS protocol elements. Despite heterogeneity in study design and outcome measurement, ERASS protocols consistently demonstrated reductions in LOS, postoperative complications, and opioid consumption.

**Table 3:** Narrative synthesis of systematic reviews reporting on Enhanced Recovery After Surgery (ERAS) protocols in spinal surgery, stratified by GRADE. This table summarises findings from studies that did not conduct a meta-analysis, integrating qualitative data and heterogeneous quantitative outcomes. Results are stratified by GRADE to reflect levels of evidence and study quality. AMP, antimicrobial prophylaxis; CBT, cognitive behavioural therapy; CI, confidence interval; ERAS, enhanced recovery after surgery; ESPB, erector spinae plane block; GRADE, Grading of Recommendations, Assessment, Development and Evaluations; LIA, local infiltration analgesia; LOS, length of stay; NSAID, non-steroidal anti-inflammatory drug; PONV, postoperative nausea and vomiting; RCTs, randomised controlled trials; SSI, surgical site infection; VAS, visual analogue scale.

	Length of stay	Postoperative complications	Cost	Pain scores	Analgesic consumption	Other
Grade I	Zaed and colleagues, 2022^30^ • ERAS protocols decreased LOS in cervical and lumbar spine surgery • Several studies evidence early oral intake hastens bowel function, reducing LOS • Early mobilisation also benefited duration of stay Liang and colleagues, 2021^27^ • EBSP had no effect on length of hospital stay	Zaed and colleagues, 2022^30^ • No difference in complications was noted amongst cervical spine surgery • Decreased complication rate was noted in studies specifically investigating lumbar spine surgery • Spinal deformity surgery with ERAS practices found reduced postoperative complications by 37%	Burgess and colleagues, 2019^23^ • Some evidence that ERAS protocols, specifically preoperative education and counselling, are able to improve healthcare expenditure	Bae and colleagues, 2022^32^ • Triple drug therapy was the most effective ERAS intervention, and reduced pain scores by 2.3 (95% CI: −3.1 to −1.4) • Double drug therapy was less effective but reduced pain scores by 1–1.6 points • Single drug therapy was largely ineffective • Graded analgesic effect noted in which analgesic efficacy increased with the number of multimodal drugs used Burgess and colleagues, 2019^23^ • Some studies found that preoperative health education and CBT do not affect lower back or leg pain scores post procedure • An alternate study found preoperative educational intervention for anxiety and pain consistently reduced VAS scores • Preoperative physiotherapy was found to reduce VAS backpain in one study Zaed and colleagues, 2022^30^ • ERAS treated patients had lower postoperative pain scores in both cervical and lumbar surgery for both deformity and degenerative disease	Bae and colleagues, 2022^32^ • Triple drug therapy was the most effective ERAS intervention, reducing morphine consumption by 26 mg (95% CI −39 to −12 mg) at 24h postoperatively. • Double drug therapy was less effective but reduced cumulative morphine consumption by 15–17 mg • Approximately 6–10 mg less 24 h morphine consumption with double therapy regimens (e.g. paracetamol + NSAID/paracetamol + adjunct/paracetamol + gabapentinoid	Burgess and colleagues, 2019^23^ • Preoperative education and counselling evidenced improved psychological outcomes (anxiety, depression and fear-avoidance), patient knowledge, feelings of better preparation, reduced negative thinking • No differences were appreciable in quality of life, return to work, physical indicators or postoperative complications associated with preoperative Zaed and colleagues, 2022^30^ • ERAS protocols in spine surgery improve many outcomes without increasing rates of readmissions
Grade II	Licina and colleagues, 2021^28^ • Prehabilitation, minimally invasive surgery, and early mobilisation were linked to reduced length of hospital stay Dietz and colleagues, 2019^22^ • Comparative reduction in LOS was reported in most studies using the ERAS protocols • Some studies found no difference between controls and ERAS protocol	Dietz and colleagues, 2019^22^ • Complication rates under ERAS protocols ranged from 2.0% to 31.7% • Decreased occurrence of adverse events with the ERAS protocol	Dietz and colleagues, 2019^22^ • Multiple studies reported reduced hospital costs with use of ERAS protocols	Licina and colleagues, 2021^28^ • Local anaesthetic wound infiltration in major spinal surgery has some immediate benefit on postoperative pain scores • Pre-admission education, pre-emptive analgesia, local anaesthetic infiltration, standard anaesthetic protocol, and perioperative analgesia were mentioned as having an impact on pain relief Dietz and colleagues, 2019^22^ • Significant pain reduction in visual analogue scale scores was observed with three ERAS protocols • Opioid-limited analgesia did not affect pain scores and reduced opioid load	Dietz and colleagues, 2019^22^ • Reduced opioid consumption after surgery, with 149.3 mg lower morphine equivalent daily dose in the ERAS protocol group compared with the non-ERAS cohort	Licina and colleagues, 2021^28^ • Postoperative fasting and PONV management were linked to improved recovery outcomes • Preoperative nutritional care, management of anaemia, and postoperative fluid and nutrition management improved recovery • Blood conservation strategies, surgical site preparation, antimicrobial prophylaxis, and thromboprophylaxis were linked to decreased blood loss and infection control Tan and colleagues, 2020^25^ • Postoperative AMP does not reduce SSI rates when pre-incisional AMP is already administered • Prolonged AMP (>48 h) offers no additional benefit over short-term AMP (<48 h) for preventing SSI • There is insufficient evidence on the duration for which wound dressings should remain intact to reduce rate of SSI in any type of spine surgery • There is insufficient evidence on the use of postoperative parenteral nutrition to reduce the rate of SSI in any type of spine surgery • The use of wound drainage systems does not impact overall SSI rates in spine surgery • No specific dressing type (e.g. negative pressure, silver-impregnated) consistently reduces SSI rates. Evidence is sparse and inconsistent • Infection-specific protocols and ERAS pathways do not significantly reduce SSI rates
Grade III	Contartese and colleagues, 2023^33^ • 81% of studies reported reduced LOS with fast-track surgery protocols • LOS was 2–5 days for spinal deformities • LOS was 2–12 days for degenerative diseases Salamanna and colleagues, 2022^31^ • ERAS protocols decreased LOS in hospitals • LOS was 1–3 days for spinal deformities • LOS was 5–10 days for degenerative diseases Tong and colleagues, 2020^24^ • Most papers concluded ERAS protocols reduced LOS, however, some found no difference when compared with traditional protocols • There was a large variation in LOS, ranging from several hours to several days Elsarrag and colleagues, 2019^10^ • ERAS protocols were found to shorten LOS and were associated with an accelerated return to function without increasing rates of complications or readmissions	Contartese and colleagues, 2023^33^ • With fast-track protocols, complication rates ranged from 1.5% to 26%. No studies reported an increase in complications associated with fast-track protocols Salamanna and colleagues, 2022^31^ • 24% of studies reported reduced postoperative complications with ERAS protocols Peng and colleagues, 2017^21^ • Multimodal analgesia incorporating gabapentin had no effect on postoperative complications	Salamanna and colleagues, 2022^31^ • 16% of studies reported reduced healthcare costs amongst ERAS protocol groups compared with traditional methods group • ERAS protocols lower costs through a number of mechanisms, by reducing complications, LOS, more efficient use of theatre resources Tong and colleagues, 2020^24^ • Multiple studies reported reduced hospital costs with use of ERAS protocols • RCTs report a cost reduction ranging from $959 to $9074 amongst ERAS groups compared with conventional protocols • Decrease in LOS and intraoperative time may translate to decreased costs	Contartese and colleagues, 2023^33^ • Significant reduction in pain through visual analogue scale (VAS) score, was observed with the fast-track protocols in 40% of studies • Multimodal pain model was consistently associated with improved pain scores • Some studies found unchanged pain scores despite decreased opioid usage Salamanna and colleagues, 2022^31^ • 24% of studies reported reduced pain scores after surgery amongst • Pain reduction via ERAS pathways were associated with preemptive analgesia, perioperative local infiltration of analgesics (LIA) and postoperative analgesia • Some studies reported unchanged pain scores, however, this correlated with reduced opioid consumption Peng and colleagues, 2017^21^ • Gabapentin analgesia significantly reduced VAS pain scores 24h after surgery, an effect that was not present 12 h post procedure	Contartese and colleagues, 2023^33^ • Multimodal opioid-sparing analgesia was able to minimise opioid burden • ERAS protocols reduced opioid usage consistently across studies • Some studies found unchanged pain scores despite decreased opioid usage Tong and colleagues, 2020^24^ • ERAS protocols consistently reduced opioid consumption after surgery • Preemptive analgesia decreased pain levels and reduced opioid consumption after surgery • Avoiding use of long-acting opioids maximises opioid sparing • Some studies reported unchanged pain scores, however, this correlated with reduced opioid consumption Peng and colleagues, 2017^21^ • Gabapentin can reduce total morphine consumption	Contartese and colleagues, 2023^33^ • Reduction in intraoperative blood loss (25%) and in transfusion rates (5%) with fast-track protocols *vs* non fast-track protocols Peng and colleagues, 2017^21^ • Multimodal analgesia with gabapentin and morphine was able to reduce urinary retention and PONV compared with traditional monomodal opioid analgesia

What is now known is that ERAS spine pathways reliably shorten LOS when they pair early oral intake with early mobilisation.^30^ LOS decreases by roughly 2–5 days for patients treated for spinal deformities and by 2–12 days in degenerative diseases,^33^ while minimally invasive spine surgery^28^ and structured prehabilitation provide additional incremental benefit. Conversely, the erector spinae plane block appears neutral^27^ for LOS and two^22 24^ SRs reported no LOS advantage when ERAS was compared with the control group. Complication data are procedure-specific: lumbar ERAS cohorts consistently show a reduction in postoperative events, whereas cervical cohorts currently show no difference, and ERASS protocols have never increased harm.^30^ Triple-drug multimodal analgesia now represents the most effective pain strategy, lowering early VAS scores by 2–3 points and cutting 24-h morphine requirements by 26 mg^32^; local anaesthetic wound infiltration offers additional analgesic gain.^28^ These improvements translate into measurable cost savings driven by shorter hospitalisation, fewer complications, and more efficient theatre time and preoperative counselling further reduces expenditure for healthcare providers.

What remains uncertain is equally clear. Long-term functional outcomes such as return-to-work, sustained quality of life, and physical rehabilitation metrics, are inconsistent, with several reviews finding no durable advantage beyond early discharge.^23^^,^^25^ Cervical, tumour-related and older cohorts remain under-represented. The additive value of infection control elements such as prolonged postoperative antibiotics, negative pressure or silver dressings, drainage systems, and parenteral nutrition is unsupported by robust evidence and often derived from small, heterogeneous studies; no dressing type consistently lowers surgical site infection rates when standard pre-incisional prophylaxis is already in place.^25^ Likewise, multimodal regimens containing gabapentin have not demonstrated clear reductions in complication rates and their impact on urinary retention or postoperative nausea and vomiting is reported in single-centre datasets only.^21^ Finally, while preoperative education improves patient anxiety, its effect on LOS, complications or opioid burden is variable and warrants further prospective study.^23^ These unresolved domains (i.e. long-term recovery, infection prevention strategies, and procedure-specific optimisation) define the next research agenda, whereas the confirmed benefits of LOS reduction, lumbar complication mitigation, and opioid stewardship justify immediate, broader adoption of core ERAS elements in spinal surgery.

## Discussion

This umbrella review synthesised findings from 17 SRs^10^^,^^21^, ^22^, ^23^, ^24^, ^25^, ^26^, ^27^, ^28^, ^29^, ^30^, ^31^, ^32^, ^33^, ^34^, ^35^, ^36^ and 55 MAs, demonstrating that ERASS protocols consistently reduce LOS, postoperative complications, opioid consumption, and healthcare costs. Specifically, ERASS protocols significantly shortened LOS by an average of 1.55 days and lowered postoperative complications by 39%. Opioid consumption was reduced by 7.26 mg (morphine equivalents) at 24 h after surgery, underscoring ERASS’s role in promoting opioid-sparing postoperative care. Additionally, healthcare costs decreased by an average of $1029.41 per patient, highlighting the economic advantages of ERASS. However, readmission rates were only reduced by 9% and patient-reported pain scores decreased by 0.27 at 24 h after surgery (recorded on a VAS or NRS 0–10 scale; i.e. 0.27 point reduction on this standard 10-point scale), both of which were non-significant.

Initially developed for colorectal surgery^1^^,^^12^ and now widely adopted across multiple surgical fields^2^, ^3^, ^4^, ^5^^,^^12^^,^^13^, ERAS protocols have shown promise in spinal surgery (only one ERAS protocol published to date, specific to lumbar spinal fusion only).^5^ Indeed, around three SRs per year have been published since 2019, reflecting growing interest in ERASS internationally. Until now, no attempt has been made to unify these efforts into a single evidence framework. In addition, no standardised ERAS guideline exists for spinal surgery overall.^5^^,^^10^

Therefore, we designed this study as an umbrella review to achieve a higher-level synthesis of the evidence. An umbrella review (i.e. a review of multiple SRs) differs from a traditional SR in that it compiles and compares the findings of published reviews, rather than re-analysing primary studies.^37^ This approach was developed in response to the growth of SRs, offering stakeholders a way to obtain an integrated overview when several reviews address overlapping topics.^37^^,^^38^ Aggregating evidence across the available ERASS-focused reviews, our umbrella review provides a comprehensive overarching perspective on perioperative enhanced recovery strategies in spinal surgery. This allows us to highlight consistent trends and resolve discrepancies among studies that would not be apparent from any single review. Umbrella reviews are now recognised as one of the highest levels of evidence synthesis^39^ and using this methodology adds distinct value by consolidating a broad evidence base into one coherent summary. Notably, we adhered to established umbrella review guidelines^37^ throughout our process, including appraisal of each included review’s quality. While an umbrella review’s conclusions inherently depend on the quality of the included SRs,^15^ this approach enabled us to synthesise the current state of ERAS in spine surgery effectively, offering clear guidance built upon the available evidence.

To capture the spectrum of impact, we deliberately investigated outcomes at three levels (reported in two or more MAs from the included 17 SRs) from a macroscopic to microscopic scale: (i) healthcare-system cost, (ii) surgical workflow (LOS, complications, readmissions), and (iii) patient-centred (pain, opioid use), thereby offering stakeholders a 360° view of ERASS benefits and trade-offs.

The included evidence spans adult and paediatric (i.e. adolescent) cohorts, elective and emergency procedures and operations ranging from single-level decompressions to complex deformity corrections; such breadth inevitably complicates statistical pooling but mirrors clinical reality. This variation likely arises from inconsistent application and reporting of ERASS components, as these protocols are often institution-specific and have not been extensively validated by larger groups. Developing a unified ERASS could help reduce variability in outcomes, supporting more reliable clinical and economic benefits across differing healthcare settings and geographic regions.

The inevitable heterogeneity of procedure type, patient mix, and ERASS elements raises a legitimate question about the trustworthiness of pooled estimates drawn from such diverse settings. We mitigated this risk in two practical ways. First, all MAs were calculated with random-effects models whenever statistical heterogeneity was present. The direction of benefit (favouring ERASS) was uniform across almost every primary cohort, strengthening the inference that the favourable signal reflects a genuine effect rather than statistical noise. Second, methodological rigour was safeguarded by subjecting every included SR to dual quality appraisal (AMSTAR-2 for conduct and ROBIS for bias) and by de-duplicating overlapping primary studies before re-analysis. Taken together, these practical measures mean that, although our pooled estimates should not be extrapolated directly to an individual operation, they do offer a reliable service delivery signal that the core principles of enhanced recovery confer net clinical and economic gains across the spine surgery landscape. In other words, in clinical practice, these results may serve as baseline evidence on which more procedure-specific ERASS pathways can be developed, tailored to anatomy, complexity, and urgency.

Figure 1 demonstrates that only 12 of the 32 ERAS elements in spinal surgery (37.5%) are examined in ≥50% of the included SRs, leaving nearly two-thirds of core items either sparsely explored or entirely absent from the current spinal ERAS literature. When we examined the interventions reported in the included SRs, several ERASS elements emerged as under-represented, especially in the absence of consensus or standard guidelines. In the preoperative stage, although patient education and counselling were almost universally adopted, blood management strategies and prophylactic antiemetic and antibiotic therapies remained sparse. During the intraoperative phase, multimodal analgesia was widely used, but details on surgical techniques, equipment choices, or minimally invasive surgery approaches were less frequently reported, possibly because of rapidly evolving evidence and the complexities of perioperative care. After surgery, interventions such as audit compliance and outcomes, patient satisfaction, postoperative glycaemic control, and structured physical therapy were least described. These disparities suggest that some ERASS components are well integrated and studied, whereas others lack sufficient emphasis or validation in the current literature. This imbalance may contribute to heterogeneity in outcomes, underscoring the need for comprehensive ERASS protocols spanning the entire perioperative continuum. Table 2 corroborates this evidence gap: merely two of the 17 SRs reported hospital cost data and only four quantified opioid consumption, despite both outcomes being priority ERAS metrics.^1^ Incorporating an additional 25 LOS cohorts, five postoperative complication cohorts, seven readmission cohorts, two hospital cost cohorts, three patient-reported pain cohorts, and seven opioid consumption cohorts that were absent from earlier syntheses, our umbrella review provides the most up-to-date pooled estimates. In doing so, it identifies high-value ERASS components that warrant prospective evaluation and offers an evidence map to help guideline panels target the understudied interventions most likely to yield further patient benefit.

In short, spine surgery teams that embed a core ERASS pathway (e.g. robust patient education, risk stratification and optimisation, antimicrobial prophylaxis, tranexamic acid therapy, maintenance of normotherapy, early mobilisation, nutrition, fluid, catheter and drain management, postoperative nausea, and vomiting prophylaxis protocol) can reasonably expect approximately 1.5 fewer inpatient days, a one-third reduction in complications, and a measurable cut in both opioid use and direct costs, even before more advanced elements are adopted. Once these elements are in place, centres can layer in next-tier elements highlighted by our gap analysis (i.e. patient blood management algorithms, perioperative glycaemic targets, structured postoperative physiotherapy, and real-time audit and feedback), which are promising but currently lack robust spine-specific data.

Several limitations were identified. The frequent use of retrospective cohorts rather than RCTs weakens the evidence and some SRs were rated low to moderate in quality, often lacking protocol registration or complete lists of excluded studies. Although deduplication procedures addressed overlap in primary studies, residual bias may persist, particularly where cost and pain outcomes varied markedly. These factors complicate drawing definitive conclusions and emphasise the need to optimise ERASS components for diverse spinal procedures. Because the 17 SRs we synthesised examined different bundles of ERAS care (some limited to a handful of preoperative measures, others encompassing the entire perioperative pathway), the evidence base is fragmented. Consequently, our umbrella review quantifies the aggregate impact of multicomponent pathways, not the isolated effect of single interventions; current meta-analytic techniques cannot report which individual element is most critical. Rigorous, element-specific trials remain essential to confirm causality and to determine the incremental value of understudied domains such as patient blood management, perioperative glycaemic control, structured postoperative physiotherapy, and audit compliance.

It is crucial to remain cautious in drawing conclusions from these findings given the modest strength of the underlying evidence. Many included studies are retrospective or observational in nature, leaving results vulnerable to bias(i.e. positive findings may be over-represented in the literature, potentially exaggerating the true effect size because of publication bias). Additionally, practice patterns differ widely across surgical settings. High-volume spine centres often already achieve substantially shorter LOS and lower complication rates through optimised standard care,^40^ and variations in healthcare systems (e.g. publicly funded *vs* private hospitals) can influence baseline outcomes.^41^ These context differences raise the possibility that ERAS’s impact, while real, might be overestimated when pooled across diverse studies. We have also noted explicitly that, because several of the contributing SRs were rated low to moderate quality on AMSTAR-2 and ROBIS, portions of our synthesis necessarily rely on lower-quality evidence, which further constrains confidence in the pooled estimates. In light of these uncertainties, a more neutral and sceptical interpretation is warranted. The benefits of ERAS are likely genuine but perhaps not as large as reported once biases and confounders are accounted for, underscoring the need for further high-quality, context-specific studies (ideally multicentre RCTs) to confirm the true magnitude of its effects.

Significant practice variations also exist across institutions and countries in managing spinal surgery,^42^ yielding different LOS, complication rates, and functional recovery.^41^^,^^43^ With no universally accepted ERAS guideline, a multidisciplinary, international working group could formalise evidence-based guidance for spinal surgery.

Moreover, for policymakers, the cost savings linked to ERASS represent a crucial opportunity to optimise healthcare resources without compromising patient care. Going forward, well designed RCTs should standardise ERASS protocols and explore under-represented elements. Through expanding this evidence base, future ERASS protocols can be tailored more effectively to different spinal procedures.

One major challenge in adopting ERASS protocols might be the inability to apply a single protocol uniformly to all operations. Therefore, tailored ERASS protocols factoring in procedure type, spinal levels, and patient comorbidities may be required. Overcoming institutional and resource barriers, enhancing staff training, and integrating cost-effectiveness analysis should also be priorities. Finally, additional research into the long-term effects of ERASS on patient-reported outcomes (i.e. quality of life, return to work, and psychological well-being) will further solidify ERASS as a comprehensive, patient-centred strategy for spine care.

### Conclusions

This umbrella review highlights the potential for ERAS protocols to improve clinical and economic outcomes in spine surgery. However, the lack of standardised guidelines and significant heterogeneity among studies underscores the need for collaborative efforts to develop and implement uniform ERASS protocols. Addressing under-represented ERASS components and standardising their application could lead to more consistent improvements in patient care, resource utilisation, and overall healthcare quality.

## Authors’ contributions

Study conception and design: DS, DD, AKD

Data acquisition, analysis and interpretation: DS, DD

Drafting the manuscript: DS, DD, AKD

Critical revision; final approval: all authors

Interpretation of data; all authors

Supervision and project administration: LS, SC, SM, SKC, ZB, STY, AKD

Provision of resources: AKD

Meet the four ICMJE authorship criteria, have approved the final version of the manuscript and agree to be accountable for all aspects of the work, ensuring that questions related to the accuracy or integrity of any part of the work are appropriately investigated and resolved: all authors.

## Declaration of interest

The authors declare that they have no conflicts of interest.
