# Supplementary material for: Optimising postoperative spine outcomes: an umbrella review of enhanced recovery after spinal surgery (ERASS) protocols
Source: Br J Anaesth. 2025 Sep 5;135(6):1663–83. doi: 10.1016/j.bja.2025.08.037 (PMC12799396; doi:10.1016/j.bja.2025.08.037)
Supplement: Multimedia Component 1 [file mmc1.pdf]

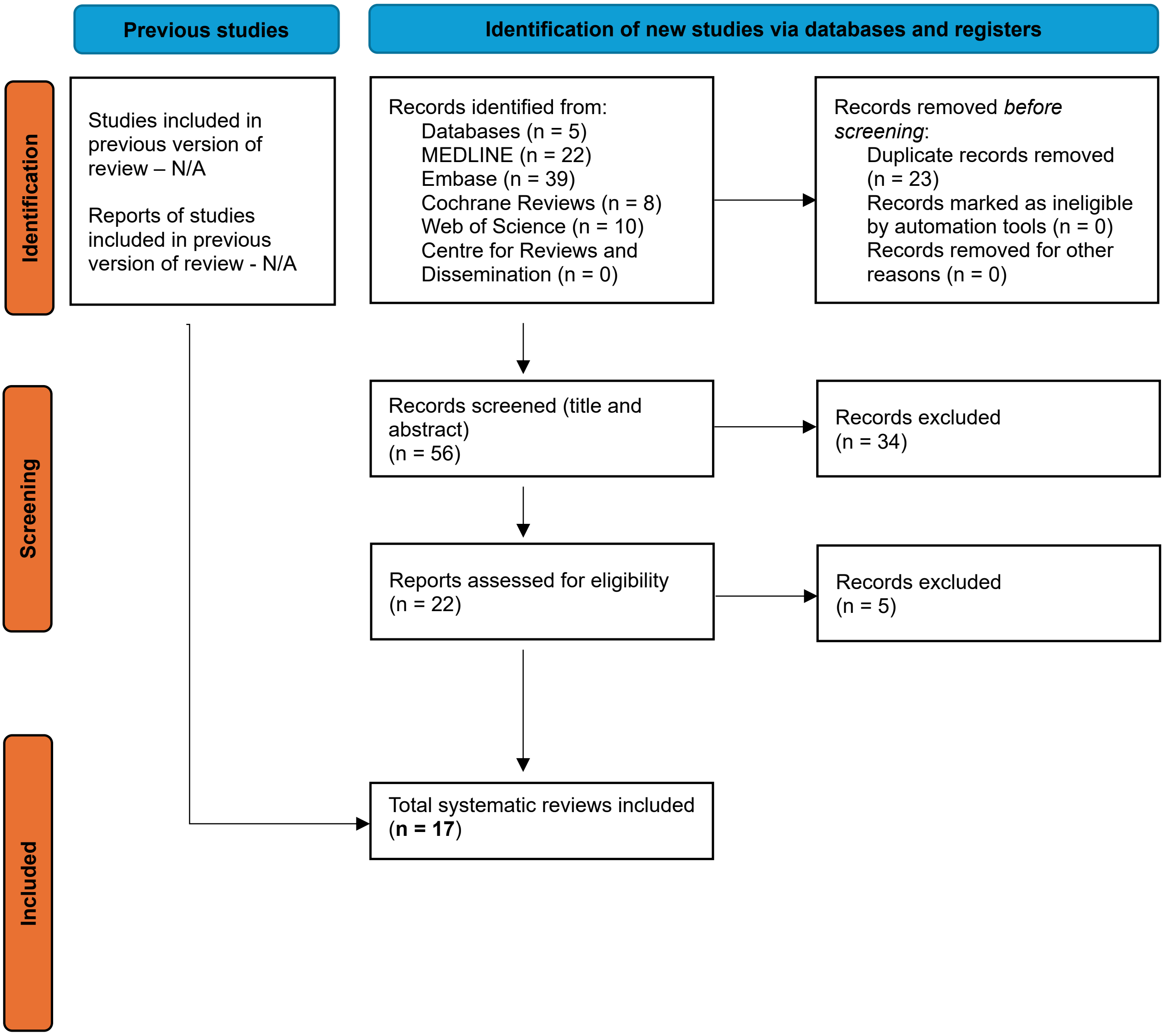

Supplementary File: Study selection flowchart (PRISMA) showing the search results and their reason for exclusion of articles.
