# Supplementary material for: Optimising postoperative spine outcomes: an umbrella review of enhanced recovery after spinal surgery (ERASS) protocols
Source: Br J Anaesth. 2025 Sep 5;135(6):1663–83. doi: 10.1016/j.bja.2025.08.037 (PMC12799396; doi:10.1016/j.bja.2025.08.037)
Supplement: Multimedia Component 2 [file mmc2.pdf]

Studies excluded from this Umbrella Review along with criteria-based reasoning

**Database**		**No. of Studies**
Medline		22
Embase		39
Web of Science		10
Cochrane Library		8
Centre for Reviews and Dissemination		0
**Total No. of Studies Identified**		**79**
**Duplicate studies**		**23**
**Titles and Abstracts Considered for Inclusion**		**56**
**Excluded Studies**		**39 Studies Excluded**
**Journal**	**Study**	**Reason Excluded**
**Medline**		**6 Excluded**
Kim H, Kim KW, Chung WS. Integrative traditional Chinese medicine for lumbar disc herniation after surgery: A protocol for systematic review and meta-analysis. Medicine (Baltimore). 2021;100(40):e27519. doi:10.1097/MD.0000000000027519		Article Type: Study Protocol not Systematic Review
Liu L, Xiao Y, Yue X, Wang Q. Safety and efficacy of enhanced recovery after surgery among patients undergoing percutaneous nephrolithotomy: a systematic review and meta-analysis. Int J Surg. 2024;110(6):3768–3777. doi:10.1097/JS9.0000000000001158		Not related to Spine surgery
Fiani B, Griepp DW, Kurien L, Adukuzhiyil J, Moawad CM, Lee J, et al. The evolving neurosurgical perspective on Enhanced Recovery After Surgery (ERAS): A systematic review. J Neurosurg Sci. 2022;66(5):391–398. doi:10.23736/S0390-5616.21.05373-X		Could not find article despite contacting the first author
Licina A, Silvers A, Laughlin H, et al. Proposed pathway for patients undergoing enhanced recovery after spinal surgery: Protocol for a systematic review. Syst Rev. 2020;9:39. doi:10.1186/s13643-020-1283-2		Article Type: Study Protocol not Systematic Review
Corniola MV, Debono B, Joswig H, Lemée JM, Tessitore E. Enhanced recovery after spine surgery: Review of the literature. Neurosurg Focus. 2019;46(4):E2. doi:10.3171/2019.1.FOCUS18657		Article Type: Comprehensive Review not Systematic Review
Kent ML, Hurley RW, Oderda GM, Gordon DB, Sun E, Mythen M, et al. American Society for Enhanced Recovery and Perioperative Quality Initiative-4 Joint Consensus Statement on Persistent Postoperative Opioid Use: Definition, incidence, risk factors, and health care system initiatives. Anesth Analg. 2019;129(2):543–552. doi:10.1213/ANE.0000000000003941		Article Type: Consensus Statement not Systematic Review

**Embase**	**18 excluded**
Kim JH, Goo B, Seo BK. Thermal stimulation with meridian points for enhanced recovery after spine surgery: A PRISMA-compliant protocol for network meta-analysis of randomized controlled trials. Medicine (Baltimore). 2023;102(22):e33909. doi:10.1097/MD.0000000000033909	Article Type: Study Protocol not Systematic Review
Pennington Z, Cottrill E, Lubelski D, Ehresman J, Lehner K, Groves ML, et al. Clinical utility of enhanced recovery after surgery pathways in pediatric spinal deformity surgery: Systematic review of the literature. J Neurosurg Pediatr. 2020;27(2):225–238. doi:10.3171/2020.7.PEDS20444	Population: Paediatric population
Tao J, Yan Z, Bai G, Zhang H, Li J. Enhanced Recovery after Surgery rehabilitation protocol in the perioperative period of orthopedics: A systematic review. J Pers Med. 2023;13(3):421. doi:10.3390/jpm13030421	Systematic Review however there was no mention of Spine surgery in the title and abstract screening
Iacob ER, Iacob R, Ghenciu LA, Popoiu TA, Stoicescu ER, Popoiu CM. Small scale, high precision: Robotic surgery in neonatal and pediatric patients—a narrative review. Children (Basel). 2024;11(3):270. doi:10.3390/children11030270	Article Type: Not Systematic Review. Paediatric Population. Not related to Spine surgery
Rizzo P, Hann H, Coombs B, Ali AAH, Stretton A, Sikander M. The Hitchhiker's Guide to Spine Awake Surgery: The Oxford SAS Protocol and early outcomes. World Neurosurg. 2023;176:e289–e296. doi:10.1016/j.wneu.2023.05.052	Article Type: Study Protocol not Systematic Review
Sethuraman MR, Murugan R, Jassim M. Letter to the editor regarding "Ultrasonography guided erector spinae block in spinal surgery for pain management with enhanced recovery." World Neurosurg X. 2023;20:100224. doi:10.1016/j.wnsx.2023.100224	Article Type: Letter to the Editor not Systematic Review
Hung KC, Chang PC, Hsu CW, Lan KM, Liao SW, Lin YT, et al. Usefulness of Analgesia Nociception Index for guiding intraoperative opioid administration: A systematic review and meta-analysis. Minerva Anestesiol. 2023;89(1-2):74–84. doi:10.23736/S0375-9393.22.16697-6	Not related to Spine surgery
Szmit M, Krajewski R, Rudnicki J, Agrawal S. Application and efficacy of transcutaneous electrical acupoint stimulation (TEAS) in clinical practice: A systematic review. Adv Clin Exp Med. 2023;32(9):1063–1074. doi:10.17219/acem/159703	Not related to Spine surgery
Fei Y, Li X. Application and prospect of enhanced recovery after surgery in patients with arthroplasty in China. Rev Assoc Med Bras (1992). 2022;68(5):697–701. doi:10.1590/1806-9282.20211136	Article Type: Not Systematic Review
Naftalovich R, Singal A, Iskander AJ. Enhanced Recovery After Surgery (ERAS) protocols for spine surgery: Review of literature. Anaesthesiol Intensive Ther. 2022;54(1):71–79. doi:10.5114/ait.2022.113961	Article Type: Comprehensive Review not Systematic Review

Laverdière C, Georgiopoulos M, Ames CP, Corban J, Ahangar P, Awadhi K, et al. Adult spinal deformity surgery and frailty: A systematic review. Glob Spine J. 2022;12(4):689–699. doi:10.1177/21925682211004250	Not related to ERAS Protocols
Ellis JL, Sudhakar A, Simhan J. Enhanced recovery strategies after penile implantation: A narrative review. Transl Androl Urol. 2021;10(6):2648–2657. doi:10.21037/tau-20-1220	Not related to Spine surgery
Blacker S, Vincent A, Burbridge M, Bustillo M, Heller B, Nadler J, et al. Spine enhanced recovery, best practice, and levels of evidence for each pathway element. J Neurosurg Anesthesiol. 2021;33(4):386–387. doi:10.1097/ANA.0000000000000800	Article Type: Conference Abstract not Systematic Review
Zileli M, Dursun E. How to improve outcomes of spine surgery in geriatric patients. World Neurosurg. 2020;140:519–526. doi:10.1016/j.wneu.2020.04.060	Article Type: Comprehensive Review not Systematic Review
Mendoza-Elias N, Whitmore RG. Commentary: Preoperative opioid use and clinical outcomes in spine surgery: A systematic review. Neurosurgery. 2020;86(6):E508. doi:10.1093/neuros/nyaa080	Article Type: Commentary not Systematic Review
Liounakos JI, Wang MY. The endoscopic approach to lumbar discectomy, fusion, and enhanced recovery: A review. Glob Spine J. 2020;10(2 Suppl):65S–69S. doi:10.1177/2192568219884913	Article Type: Comprehensive Review not Systematic Review
Burgess L, et al. The inclusion of preoperative education in ERAS spinal surgery pathways: A systematic review. Clin Nutr ESPEN. 2019;31:107.	Article Type: Conference Abstract not Systematic Review
Deng QF, Gu HY, Peng WY, Zhang Q, Huang ZD, Zhang C, et al. Impact of enhanced recovery after surgery on postoperative recovery after joint arthroplasty: Results from a systematic review and meta-analysis. Postgrad Med J. 2018;94(1118):678–693. doi:10.1136/postgradmedj-2018-136166	Not related to Spine surgery
**Web of Science**	**7 Excluded**
Debono B, Wainwright TW, Wang MY, Sigmundsson FG, Yang MMH, Smid-Nanninga H, et al. Consensus statement for perioperative care in lumbar spinal fusion: Enhanced Recovery After Surgery (ERAS®) Society recommendations. Spine J. 2021;21(5):729–752. doi:10.1016/j.spinee.2021.01.001	Article Type: Consensus Paper not Systematic Review
Greene R, Furlong B, Smith-Forrester J, Swab M, Christie S, Etchegary H, et al. The effect of Enhanced Recovery After Surgery protocols for elective cervical and lumbar spine procedures on hospital length of stay: A systematic review and meta-analysis. Int J Behav Med. 2023;30(0):S160. doi:10.1007/s12529-023-13345-4 (System ID: 13277454).	Could not find article despite contacting the first author

Wallström A, Frisman GH. Facilitating early recovery of bowel motility after colorectal surgery: A systematic review. J Clin Nurs. 2014;23(1-2):24–44. doi:10.1111/jocn.12258	Article Type: Comprehensive Review not Systematic Review. Not related to Spine surgery.
Bradko V, Castillo H, Janardhan S, Dahl B, Gandy K, Castillo J. Towards guideline-based management of tethered cord syndrome in spina bifida: A global health paradigm shift in the era of prenatal surgery. Neurospine. 2019;16(4):715–727. doi:10.14245/ns.1836342.171	Population: Paediatric population. Not related to ERAS Protocols.
Teja BJ, Sutherland TN, Barnett SR, Talmor DS. Cost-effectiveness research in anesthesiology. Anesth Analg. 2018;127(5):1196–1201. doi:10.1213/ANE.0000000000003334	Not related to Spine surgery. Not related to ERAS Protocols.
Kim SW. The increase of yellow dust records during the Wei-Jin-Nan-Bei-Chao era: Causes and environmental history analysis. J Ecol Environ Hist. 2022;8(0):117–169. doi:System ID: 13277460.	Not related to Spine surgery. Not related to ERAS Protocols.
Neves JFPC. Functional impact after lumbar arthrodesis: Systematic literature review. [Book]. 2019;0(0):[no pagination]. System ID: 13277461.	Article Type: Literature Review not Systematic Review
**Cochrane Library**	**8 Excluded**
Palacios P, Palacios I, Palacios A, Gutiérrez JC, Mariscal G, Lorente A. Efficacy and safety of the extreme lateral interbody fusion (XLIF) technique in spine surgery: meta-analysis of 1409 patients. J Clin Med. 2024;13(4):960. doi:10.3390/jcm13040960	Article Type: Clinical Trial not Systematic Review
Hepworth EP, Lee A, Pardo Pardo J, Aydin SZ, Tugwell P. Short-term induction glucocorticoids and disease-modifying anti-rheumatic drugs (DMARD) therapy for rheumatoid arthritis. Cochrane Database Syst Rev. 2021;2021(12):CD014897. doi:10.1002/14651858.CD014897	Article Type: Study Protocol not Systematic Review & Not related to Spine surgery
France E, Uny I, Turley R, Thomson K, Noyes J, Jordan A, et al. A meta-ethnography of how children and young people with chronic non-cancer pain and their families experience and understand their condition, pain services, and treatments. Cochrane Database Syst Rev. 2023;10(10):CD014873. doi:10.1002/14651858.CD014873.pub2	Article Type: Review not Systematic Review
Matthews E, Brassington R, Kuntzer T, Jichi F, Manzur AY. Corticosteroids for the treatment of Duchenne muscular dystrophy. Cochrane Database Syst Rev. 2016;2016(5):CD003725. doi:10.1002/14651858.CD003725.pub4	Not related to Spine surgery
Weinstein EJ, Levene JL, Cohen MS, Andreae DA, Chao JY, Johnson M, et al. Local anaesthetics and regional anaesthesia versus conventional analgesia for preventing persistent postoperative pain in adults and children. Cochrane Database Syst Rev. 2018;4(4):CD007105. doi:10.1002/14651858.CD007105.pub3	Systematic Review however there was no mention of Spine surgery in the title and abstract screening

Smith MD, McCall J, Plank L, Herbison GP, Soop M, Nygren J. Preoperative carbohydrate treatment for enhancing recovery after elective surgery. Cochrane Database Syst Rev. 2014;2014(8):CD009161. doi:10.1002/14651858.CD009161.pub2	Systematic Review however there was no mention of Spine surgery in the title and abstract screening
Weibel S, Jelting Y, Pace NL, Helf A, Eberhart LH, Hahnenkamp K, et al. Continuous intravenous perioperative lidocaine infusion for postoperative pain and recovery in adults. Cochrane Database Syst Rev. 2018;6(6):CD009642. doi:10.1002/14651858.CD009642.pub3	Systematic Review however there was no mention of Spine surgery in the title and abstract screening
Oostvogels L, Weibel S, Meißner M, Kranke P, Meyer-Frießem CH, Pogatzki-Zahn E, et al. Erector spinae plane block for postoperative pain. Cochrane Database Syst Rev. 2024;2(2):CD013763. doi:10.1002/14651858.CD013763.pub3	Systematic Review however there was no mention of Spine surgery in the title and abstract screening
**Studies included in the Umbrella Review**	**17 Included**
