# Supplementary material for: Optimising postoperative spine outcomes: an umbrella review of enhanced recovery after spinal surgery (ERASS) protocols
Source: Br J Anaesth. 2025 Sep 5;135(6):1663–83. doi: 10.1016/j.bja.2025.08.037 (PMC12799396; doi:10.1016/j.bja.2025.08.037)
Supplement: Multimedia Component 3 [file mmc3.pdf]

References of Primary Studies included in this Umbrella Review (319 Studies)

1. Adeyemo EA, Aoun SG, Barrie U, Nguyen ML, Badejo O, Pernik MN, et al. Enhanced Recovery After Surgery Reduces Postoperative Opioid Use and 90-Day Readmission Rates After Open Thoracolumbar Fusion for Adult Degenerative Deformity. Neurosurgery. 2021;88(2):295-300.
2. Adeyemo EA, Aoun SG, Barrie U, Nguyen ML, Johnson ZD, Hall K, et al. Comparison of the effect of epidural versus intravenous patient controlled analgesia on inpatient and outpatient functional outcomes after adult degenerative scoliosis surgery: a comparative study. Spine J. 2021;21(5):765-71.
3. Adogwa O, Carr K, Thompson P, Hoang K, Darlington T, Perez E, et al. A prospective, multi-institutional comparative effectiveness study of lumbar spine surgery in morbidly obese patients: does minimally invasive transforaminal lumbar interbody fusion result in superior outcomes? World Neurosurg. 2015;83(5):860-6.
4. Adogwa O, Martin JR, Huang K, Verla T, Fatemi P, Thompson P, et al. Preoperative serum albumin level as a predictor of postoperative complication after spine fusion. Spine (Phila Pa 1976). 2014;39(18):1513-9.
5. Aglio LS, Abd-El-Barr MM, Orhurhu V, Kim GY, Zhou J, Gugino LD, et al. Preemptive analgesia for postoperative pain relief in thoracolumbosacral spine operations: a double-blind, placebo-controlled randomized trial. J Neurosurg Spine. 2018;29(6):647-53.
6. Aiyer SN, Kumar A, Shetty AP, Kanna RM, Rajasekaran S. Factors Influencing Postoperative Urinary Retention Following Elective Posterior Lumbar Spine Surgery: A Prospective Study. Asian Spine J. 2018;12(6):1100-5.
7. Alajmi T, Saeed H, Alfaryan K, Alakeel A, Alfaryan T. Efficacy of tranexamic acid in reducing blood loss and blood transfusion in idiopathic scoliosis: a systematic review and meta-analysis. J Spine Surg. 2017;3(4):531-40.
8. Ali ZS, Albayar A, Nguyen J, Gallagher RS, Borja AJ, Kallan MJ, et al. A Randomized Controlled Trial to Assess the Impact of Enhanced Recovery After Surgery on Patients Undergoing Elective Spine Surgery. Annals of surgery. 2023;278(3):408-16.
9. Ali ZS, Flanders TM, Ozturk AK, Malhotra NR, Leszinsky L, McShane BJ, et al. Enhanced recovery after elective spinal and peripheral nerve surgery: pilot study from a single institution. Journal of neurosurgery Spine. 2019;30(4):532-40.
10. AlSaleh K, Murrad K, AlZakri A, Alrehaili O, Awwad W. Enhanced Recovery Pathway in Adults Undergoing Elective Posterior Thoracolumbar Fusion Surgery: Outcomes Compared with a Traditional Care Pathway. Adv Orthop. 2021;2021:6204831-.
11. Altschul D, Kobets AJ, Nakhla J, Jada A, Nasser R, Kinon MD, et al. Postoperative urinary retention in patients undergoing elective spinal surgery. Journal of neurosurgery Spine. 2017;26(2):229-34.
12. Andersen T, Christensen FB, Laursen M, Høy K, Hansen ES, Bünger C. Smoking as a predictor of negative outcome in lumbar spinal fusion. Spine (Phila Pa 1976). 2001;26(23):2623-8.
13. Angus M, Jackson K, Smurthwaite G, Carrasco R, Mohammad S, Verma R, et al. The implementation of enhanced recovery after surgery (ERAS) in complex spinal surgery. Journal of spine surgery (Hong Kong). 2019;5(1):116-23.
14. Armaghani SJ, Archer KR, Rolfe R, Demaio DN, Devin CJ. Diabetes Is Related to Worse Patient-Reported Outcomes at Two Years Following Spine Surgery. J Bone Joint Surg Am. 2016;98(1):15-22.

15. Asar S, Sarı S, Altinpulluk EY, Turgut M. Efficacy of erector spinae plane block on postoperative pain in patients undergoing lumbar spine surgery. European Spine Journal. 2022;31(1):197-204.
16. Attia JZ, Mansour HS. Perioperative Duloxetine and Etoricoxibto improve postoperative pain after lumbar Laminectomy: a randomized, double-blind, controlled study. BMC Anesthesiol. 2017;17(1):162.
17. Aubrun F, Langeron O, Heitz D, Coriat P, Riou B. Randomised, placebo-controlled study of the postoperative analgesic effects of ketoprofen after spinal fusion surgery. Acta Anaesthesiol Scand. 2000;44(8):934-9.
18. Aveline C, Hetet HL, Vautier P, Gautier JF, Bonnet F. Peroperative ketamine and morphine for postoperative pain control after lumbar disk surgery. European Journal of Pain. 2006;10(7):653-8.
19. Avni T, Bieber A, Grossman A, Green H, Leibovici L, Gafter-Gvili A. The safety of intravenous iron preparations: systematic review and meta-analysis. Mayo Clin Proc. 2015;90(1):12-23.
20. Awad S, Varadhan KK, Ljungqvist O, Lobo DN. A meta-analysis of randomised controlled trials on preoperative oral carbohydrate treatment in elective surgery. Clinical Nutrition. 2013;32(1):34-44.
21. Bacchin MR, Ceria CM, Giannone S, Ghisi D, Stagni G, Greggi T, et al. Goal-Directed Fluid Therapy Based on Stroke Volume Variation in Patients Undergoing Major Spine Surgery in the Prone Position: A Cohort Study. Spine (Phila Pa 1976). 2016;41(18):E1131-e7.
22. Band IC, Yenicay AO, Montemurno TD, Chan JS, Ogden AT. Enhanced Recovery After Surgery Protocol in Minimally Invasive Lumbar Fusion Surgery Reduces Length of Hospital Stay and Inpatient Narcotic Use. World Neurosurg X. 2022;14:100120.
23. Bansal T, Sharan AD, Garg B. Enhanced recovery after surgery (ERAS) protocol in spine surgery. J Clin Orthop Trauma. 2022;31:101944-.
24. Barker FG, 2nd. Efficacy of prophylactic antibiotic therapy in spinal surgery: a meta-analysis. Neurosurgery. 2002;51(2):391-400; discussion -1.
25. Batko I, Kościelniak-Merak B, Tomasik PJ, Kobylarz K, Wordliczek J. Lidocaine as an element of multimodal analgesic therapy in major spine surgical procedures in children: a prospective, randomized, double-blind study. Pharmacol Rep. 2020;72(3):744-55.
26. Bedin A, Caldart Bedin RA, Vieira JE, Ashmawi HA. Duloxetine as an Analgesic Reduces Opioid Consumption After Spine Surgery: A Randomized, Double-Blind, Controlled Study. Clin J Pain. 2017;33(10):865-9.
27. Bednar DA. Description and Results of a Comprehensive Care Protocol for Overnight-Stay Spine Surgery in Adults. Spine (Phila Pa 1976). 2017;42(14):E871-e5.
28. Blitz JD, Kendale SM, Jain SK, Cuff GE, Kim JT, Rosenberg AD. Preoperative Evaluation Clinic Visit Is Associated with Decreased Risk of In-hospital Postoperative Mortality. Anesthesiology. 2016;125(2):280-94.
29. Boenigk K, Echevarria GC, Nisimov E, von Bergen Granell AE, Cuff GE, Wang J, et al. Low-dose ketamine infusion reduces postoperative hydromorphone requirements in opioid-tolerant patients following spinal fusion: A randomised controlled trial. Eur J Anaesthesiol. 2019;36(1):8-15.
30. Boezaart AP, Eksteen JA, Spuy GVD, Rossouw P, Knipe M. Intrathecal morphine. Double-blind evaluation of optimal dosage for analgesia after major lumbar spinal surgery. Spine. 1999;24(11):1131-7.

31. Bohl DD, Louie PK, Shah N, Mayo BC, Ahn J, Kim TD, et al. Multimodal Versus Patient-Controlled Analgesia After an Anterior Cervical Decompression and Fusion. Spine. 2016;41(12):994-8.
32. Bowen RE, Gardner S, Scaduto AA, Eagan M, Beckstead J. Efficacy of intraoperative cell salvage systems in pediatric idiopathic scoliosis patients undergoing posterior spinal fusion with segmental spinal instrumentation. Spine (Phila Pa 1976). 2010;35(2):246-51.
33. Bradywood A, Farrokhi F, Williams B, Kowalczyk M, Blackmore CC. Reduction of Inpatient Hospital Length of Stay in Lumbar Fusion Patients With Implementation of an Evidence-Based Clinical Care Pathway. Spine (Phila Pa 1976). 2017;42(3):169-76.
34. Brusko GD, Kolcun JPG, Heger JA, Levi AD, Manzano GR, Madhavan K, et al. Reductions in length of stay, narcotics use, and pain following implementation of an enhanced recovery after surgery program for 1- to 3-level lumbar fusion surgery. Neurosurg Focus. 2019;46(4):E4-E.
35. Bydon M, De la Garza-Ramos R, Abt NB, Gokaslan ZL, Wolinsky JP, Sciubba DM, et al. Impact of smoking on complication and pseudarthrosis rates after single- and 2-level posterolateral fusion of the lumbar spine. Spine (Phila Pa 1976). 2014;39(21):1765-70.
36. Cakan T, Inan N, Culhaoglu S, Bakkal K, Başar H. Intravenous paracetamol improves the quality of postoperative analgesia but does not decrease narcotic requirements. J Neurosurg Anesthesiol. 2008;20(3):169-73.
37. Calia R, La Brocca L, Ventimiglia M, Favaro G, Catania Cucchiara T, Marino C, et al. ESRA19-0353 The erector spinae plane block as an analgesic regional technique in acute post-surgical pain control in lumbar surgery. Preliminary findings of a randomized trial. Regional Anesthesia & Pain Medicine. 2019;44(Suppl 1):A237.
38. Carr DA, Saigal R, Zhang F, Bransford RJ, Bellabarba C, Dagal A. Enhanced perioperative care and decreased cost and length of stay after elective major spinal surgery. Neurosurg Focus. 2019;46(4):E5-E.
39. Chadduck JB, Sneyd JR, Pobereskin LH. The role of bupivacaine in early postoperative pain control after lumbar decompression. J Neurosurg. 1999;90(1 Suppl):67-72.
40. Chakravarthy VB, Laufer I, Amin AG, Cohen MA, Reiner AS, Vuong C, et al. Patient outcomes following implementation of an enhanced recovery after surgery pathway for patients with metastatic spine tumors. Cancer. 2022;128(23):4109-18.
41. Chakravarthy VB, Yokoi H, Coughlin DJ, Manlapaz MR, Krishnaney AA. Development and implementation of a comprehensive spine surgery enhanced recovery after surgery protocol: the Cleveland Clinic experience. Neurosurgical focus. 2019;46(4).
42. Chan CYW, Loo SF, Ong JY, Lisitha KA, Hasan MS, Lee CK, et al. Feasibility and Outcome of an Accelerated Recovery Protocol in Asian Adolescent Idiopathic Scoliosis Patients. Spine. 2017;42(24):E1415-E22.
43. Chang HK, Huang M, Wu JC, Huang WC, Wang MY. Less Opioid Consumption With Enhanced Recovery After Surgery Transforaminal Lumbar Interbody Fusion (TLIF): A Comparison to Standard Minimally-Invasive TLIF. Neurospine. 2020;17(1):228-36.
44. Chang WK, Wu HL, Yang CS, Chang KY, Liu CL, Chan KH, et al. Effect on pain relief and inflammatory response following addition of tenoxicam to intravenous patient-controlled morphine analgesia: a double-blind, randomized, controlled study in patients undergoing spine fusion surgery. Pain Med. 2013;14(5):736-48.
45. Chen J, Li D, Wang R, Wang S, Shang Z, Wang M, et al. Benefits of the Enhanced Recovery After Surgery Program in Short-Segment Posterior Lumbar Interbody Fusion Surgery. World Neurosurg. 2022;159:e303-e10.

46. Chen K, Wang L, Liu X, Lu Y. Ultrasound-Guided Erector Spinae Plane Block Reduces Perioperative Opioid Consumption in Lumbar Spinal Fusion. American Journal of Therapeutics. 2021;28(2):e266-e8.
47. Chen K, Wang L, Ning M, Dou L, Li W, Li Y. Evaluation of ultrasound-guided lateral thoracolumbar interfascial plane block for postoperative analgesia in lumbar spine fusion surgery: a prospective, randomized, and controlled clinical trial. PeerJ. 2019;7:e7967.
48. Chen W, Yang H, Jiang X, Fan S. The Effect of Oblique Lumbar Interbody Fusion Compared with Transforaminal Lumbar Interbody Fusion Combined with Enhanced Recovery after Surgery Program on Patients with Lumbar Degenerative Disease at Short-Term Follow-Up. Biomed Res Int. 2021;2021:5806066.
49. Chen Y. The practice and efect eval- uation of target management in the rapid recovery of patients with anterior cervical spine surgery. Healthcare. 2019.
50. Chin KR, Coombs AV, Seale JA. Feasibility and patient-reported outcomes after outpatient single-level instrumented posterior lumbar interbody fusion in a surgery center: preliminary results in 16 patients. Spine (Phila Pa 1976). 2015;40(1):E36-42.
51. Choi HY, Hyun SJ, Kim KJ, Jahng TA, Kim HJ. Clinical Efficacy of Intra-Operative Cell Salvage System in Major Spinal Deformity Surgery. J Korean Neurosurg Soc. 2019;62(1):53-60.
52. Choi YS, Shim JK, Song JW, Kim JC, Yoo YC, Kwak YL. Combination of pregabalin and dexamethasone for postoperative pain and functional outcome in patients undergoing lumbar spinal surgery: a randomized placebo-controlled trial. Clin J Pain. 2013;29(1):9-14.
53. Chuang MF, Tung HH, Clinciu DL, Huang JS, Iqbal U, Chang CJ, et al. The effect of an integrated education model on anxiety and uncertainty in patients undergoing cervical disc herniation surgery. Comput Methods Programs Biomed. 2016;133:17-23.
54. Ciftci B, Ekinci M, Celik EC, Yayik AM, Aydin ME, Ahiskalioglu A. Ultrasound-Guided Erector Spinae Plane Block versus Modified-Thoracolumbar Interfascial Plane Block for Lumbar Discectomy Surgery: A Randomized, Controlled Study. World Neurosurg. 2020;144:e849-e55.
55. Cohen BE, Hartman MB, Wade JT, Miller JS, Gilbert R, Chapman TM. Postoperative pain control after lumbar spine fusion. Patient-controlled analgesia versus continuous epidural analgesia. Spine (Phila Pa 1976). 1997;22(16):1892-6; discussion 6-7.
56. Corcoran T, Rhodes JE, Clarke S, Myles PS, Ho KM. Perioperative fluid management strategies in major surgery: a stratified meta-analysis. Anesth Analg. 2012;114(3):640-51.
57. Crawford MW, Hickey C, Zaarour C, Howard A, Naser B. Development of acute opioid tolerance during infusion of remifentanil for pediatric scoliosis surgery. Anesth Analg. 2006;102(6):1662-7.
58. Cui P, Wang S, Wang P, Yang L, Kong C, Lu S. Comparison of perioperative outcomes in frail patients following multilevel lumbar fusion surgery with and without the implementation of the enhanced recovery after surgery protocol. Frontiers in Surgery. 2022;9.
59. Curley KL, Richards AE, Zhang N, Lyons MK, Neal MT. Enhanced recovery after posterior cervical fusion surgery: A retrospective case series. Interdisciplinary Neurosurgery. 2021;25:101143-.
60. d'Astorg H, Fière V, Dupasquier M, Vieira TD, Szadkowski M. Enhanced recovery after surgery (ERAS) protocol reduces LOS without additional adverse events in spine surgery. Orthop Traumatol Surg Res. 2020;106(6):1167-73.
61. Dagal A, Bellabarba C, Bransford R, Zhang F, Chesnut RM, O'Keefe GE, et al. Enhanced Perioperative Care for Major Spine Surgery. Spine (Phila Pa 1976). 2019;44(13):959-66.

62. Dai B, Gao P, Dong QR, Wang YM, Chen D, Shen YC, et al. [Clinical study of the application of enhanced recovery after surgery in cervical spondylotic myelopathy]. Zhongguo Gu Shang. 2018;31(8):740-5.
63. Debono B, Corniola MV, Pietton R, Sabatier P, Hamel O, Tessitore E. Benefits of Enhanced Recovery After Surgery for fusion in degenerative spine surgery: impact on outcome, length of stay, and patient satisfaction. Neurosurg Focus. 2019;46(4):E6.
64. Debono B, Sabatier P, Boniface G, Bousquet P, Lescure JP, Garnaud V, et al. Implementation of enhanced recovery after surgery (ERAS) protocol for anterior cervical discectomy and fusion: a propensity score-matched analysis. European spine journal : official publication of the European Spine Society, the European Spinal Deformity Society, and the European Section of the Cervical Spine Research Society. 2021;30(2):560-7.
65. Dehkordy ME, Tavanaei R, Younesi E, Khorasanizade S, Farsani HA, Oraee-Yazdani S. Effects of perioperative magnesium sulfate infusion on intraoperative blood loss and postoperative analgesia in patients undergoing posterior lumbar spinal fusion surgery: A randomized controlled trial. Clin Neurol Neurosurg. 2020;196:105983.
66. Deng QF, Gu HY, Peng WY, Zhang Q, Huang ZD, Zhang C, et al. Impact of enhanced recovery after surgery on postoperative recovery after joint arthroplasty: results from a systematic review and meta-analysis. Postgrad Med J. 2018;94(1118):678-93.
67. Derby R, Lettice JJ, Kula TA, Lee SH, Seo KS, Kim BJ. Single-level lumbar fusion in chronic discogenic low-back pain: psychological and emotional status as a predictor of outcome measured using the 36-item Short Form. J Neurosurg Spine. 2005;3(4):255-61.
68. DeVries Z, Barrowman N, Smit K, Mervitz D, Moroz P, Tice A, et al. Is it feasible to implement a rapid recovery pathway for adolescent idiopathic scoliosis patients undergoing posterior spinal instrumentation and fusion in a single-payer universal health care system? Spine Deform. 2020;8(6):1223-9.
69. Dhaliwal P, Yavin D, Whittaker T, Hawboldt GS, Jewett GAE, Casha S, et al. Intrathecal Morphine Following Lumbar Fusion: A Randomized, Placebo-Controlled Trial. Neurosurgery. 2019;85(2):189-98.
70. Ding G. Effect of fast-track surgery concept on perioperative nursing care of patients undergoing anterior cervical spine surgery. Journal of Huaihai Medicine. 2021.
71. Djordjevic Z, Jankovic S, Gajovic O, Djonovic N, Folic N, Bukumiric Z. Hospital infections in a neurological intensive care unit: incidence, causative agents and risk factors. J Infect Dev Ctries. 2012;6(11):798-805.
72. Duojun W, Hui Z, Zaijun L, Yuxiang G, Haihong C. Enhanced recovery after surgery pathway reduces the length of hospital stay without additional complications in lumbar disc herniation treated by percutaneous endoscopic transforaminal discectomy. J Orthop Surg Res. 2021;16(1):461.
73. Eckman WW, Hester L, McMillen M. Same-day discharge after minimally invasive transforaminal lumbar interbody fusion: a series of 808 cases. Clin Orthop Relat Res. 2014;472(6):1806-12.
74. Eiamcharoenwit J, Chotisukarat H, Tainil K, Attanath N, Akavipat P. Analgesic efficacy of intravenous nefopam after spine surgery: a randomized, double-blind, placebo-controlled trial. F1000Res. 2020;9:516.
75. Elgamal SM, Abdelhalim AA, Arida EA, Elhabashy AM, Sabra RAE. Enhanced recovery after spinal surgery protocol versus conventional care in non- insulin diabetic patients: A prospective randomized trial. Egyptian Journal of Anaesthesia. 2023;39(1):313-21.

76. Elia N, Lysakowski C, Tramèr MR. Does multimodal analgesia with acetaminophen, nonsteroidal antiinflammatory drugs, or selective cyclooxygenase-2 inhibitors and patient-controlled analgesia morphine offer advantages over morphine alone? Meta-analyses of randomized trials. Anesthesiology. 2005;103(6):1296-304.
77. Elsamadicy AA, Adogwa O, Vuong VD, Mehta AI, Vasquez RA, Cheng J, et al. Association of Intraoperative Blood Transfusions on Postoperative Complications, 30-Day Readmission Rates, and 1-Year Patient-Reported Outcomes. Spine (Phila Pa 1976). 2017;42(8):610-5.
78. Emir E, Serin S, Erbay RH, Sungurtekin H, Tomatir E. Tramadol versus low dose tramadol-paracetamol for patient controlled analgesia during spinal vertebral surgery. Kaohsiung J Med Sci. 2010;26(6):308-15.
79. Ersayli DT, Gurbet A, Bekar A, Uckunkaya N, Bilgin H. Effects of perioperatively administered bupivacaine and bupivacaine-methylprednisolone on pain after lumbar discectomy. Spine (Phila Pa 1976). 2006;31(19):2221-6.
80. Eskin MB, Ceylan A, Özhan M, Atik B. Ultrasound-guided erector spinae block versus mid-transverse process to pleura block for postoperative analgesia in lumbar spinal surgery. Anaesthesist. 2020;69(10):742-50.
81. Esmail F, Mohammad-Reza F, Homayoon T. Preemptive analgesia with local lidocaine infiltration for single-level open disc operation. Pak J Biol Sci. 2008;11(14):1868-71.
82. Ezhevskaya AA, Ovechkin AM, Prusakova ZB, Zagrekov VI, Mlyavykh SG, Anderson DG. Relationship among anesthesia technique, surgical stress, and cognitive dysfunction following spinal surgery: a randomized trial. J Neurosurg Spine. 2019;31(6):894-901.
83. Farag E, Ghobrial M, Sessler DI, Dalton JE, Liu J, Lee JH, et al. Effect of perioperative intravenous lidocaine administration on pain, opioid consumption, and quality of life after complex spine surgery. Anesthesiology. 2013;119(4):932-40.
84. Farhad E, Mansour F, Hamid Reza S, Maysam A, Mohammad Reza K. The Effect of Intraoperative Ketamine and Magnesium Sulfate on Acute Pain and Opioid Consumption After Spine Surgery. Acta Medica Iranica. 2020;58(5).
85. Farmery AD, Wilson-MacDonald J. The analgesic effect of epidural clonidine after spinal surgery: a randomized placebo-controlled trial. Anesth Analg. 2009;108(2):631-4.
86. Feng C, Zhang Y, Chong F, Yang M, Liu C, Liu L, et al. Establishment and Implementation of an Enhanced Recovery After Surgery (ERAS) Pathway Tailored for Minimally Invasive Transforaminal Lumbar Interbody Fusion Surgery. World Neurosurg. 2019;129:e317-e23.
87. Finnerty D, A NE, Ahmed M, Poynton A, Butler JS, Buggy DJ. A randomised trial of bilateral erector spinae plane block vs. no block for thoracolumbar decompressive spinal surgery. Anaesthesia. 2021;76(11):1499-503.
88. Firouzian A, Gholipour Baradari A, Alipour A, Emami Zeydi A, Zamani Kiasari A, Emadi SA, et al. Ultra-low-dose Naloxone as an Adjuvant to Patient Controlled Analgesia (PCA) With Morphine for Postoperative Pain Relief Following Lumber Discectomy: A Double-blind, Randomized, Placebo-controlled Trial. J Neurosurg Anesthesiol. 2018;30(1):26-31.
89. Flanders TM, Ifrach J, Sinha S, Joshi DS, Ozturk AK, Malhotra NR, et al. Reduction of Postoperative Opioid Use After Elective Spine and Peripheral Nerve Surgery Using an Enhanced Recovery After Surgery Program. Pain Med. 2020;21(12):3283-91.
90. Fletcher ND, Andras LM, Lazarus DE, Owen RJ, Geddes BJ, Cao J, et al. Use of a Novel Pathway for Early Discharge Was Associated With a 48% Shorter Length of Stay After Posterior Spinal Fusion for Adolescent Idiopathic Scoliosis. J Pediatr Orthop. 2017;37(2):92-7.

91. Fletcher ND, Bellaire LL, Dilbone ES, Ward LA, Bruce RW, Jr. Variability in length of stay following neuromuscular spinal fusion. Spine Deform. 2020;8(4):725-32.
92. Fletcher ND, Murphy JS, Austin TM, Bruce RW, Jr., Harris H, Bush P, et al. Short term outcomes of an enhanced recovery after surgery (ERAS) pathway versus a traditional discharge pathway after posterior spinal fusion for adolescent idiopathic scoliosis. Spine Deform. 2021;9(4):1013-9.
93. Fletcher ND, Shourbaji N, Mitchell PM, Oswald TS, Devito DP, Bruce RW. Clinical and economic implications of early discharge following posterior spinal fusion for adolescent idiopathic scoliosis. J Child Orthop. 2014;8(3):257-63.
94. France JC, Jorgenson SS, Lowe TG, Dwyer AP. The use of intrathecal morphine for analgesia after posterolateral lumbar fusion: a prospective, double-blind, randomized study. Spine. 1997;22(19):2272-7.
95. Fu MC, Buerba RA, Grauer JN. Preoperative Nutritional Status as an Adjunct Predictor of Major Postoperative Complications Following Anterior Cervical Discectomy and Fusion. Clin Spine Surg. 2016;29(4):167-72.
96. Fujita N, Tobe M, Tsukamoto N, Saito S, Obata H. A randomized placebo-controlled study of preoperative pregabalin for postoperative analgesia in patients with spinal surgery. J Clin Anesth. 2016;31:149-53.
97. Gande A, Rosinski A, Cunningham T, Bhatia N, Lee YP. Selection pressures of vancomycin powder use in spine surgery: a meta-analysis. Spine J. 2019;19(6):1076-84.
98. Garg B, Mehta N, Bansal T, Shekhar S, Khanna P, Baidya DK. Design and Implementation of an Enhanced Recovery After Surgery Protocol in Elective Lumbar Spine Fusion by Posterior Approach: A Retrospective, Comparative Study. Spine (Phila Pa 1976). 2021;46(12):E679-e87.
99. Garg N, Panda NB, Gandhi KA, Bhagat H, Batra YK, Grover VK, et al. Comparison of Small Dose Ketamine and Dexmedetomidine Infusion for Postoperative Analgesia in Spine Surgery--A Prospective Randomized Double-blind Placebo Controlled Study. J Neurosurg Anesthesiol. 2016;28(1):27-31.
100. Gause PR, Siska PA, Westrick ER, Zavatsky J, Irrgang JJ, Kang JD. Efficacy of intraoperative cell saver in decreasing postoperative blood transfusions in instrumented posterior lumbar fusion patients. Spine (Phila Pa 1976). 2008;33(5):571-5.
101. Ghobrial GM, Wang MY, Green BA, Levene HB, Manzano G, Vanni S, et al. Preoperative skin antisepsis with chlorhexidine gluconate versus povidone-iodine: a prospective analysis of 6959 consecutive spinal surgery patients. J Neurosurg Spine. 2018;28(2):209-14.
102. Gianesello L, Pavoni V, Barboni E, Galeotti I, Nella A. Perioperative pregabalin for postoperative pain control and quality of life after major spinal surgery. J Neurosurg Anesthesiol. 2012;24(2):121-6.
103. Glassman SD, Anagnost SC, Parker A, Burke D, Johnson JR, Dimar JR. The effect of cigarette smoking and smoking cessation on spinal fusion. Spine (Phila Pa 1976). 2000;25(20):2608-15.
104. Goel VK, Chandramohan M, Murugan C, Shetty AP, Subramanian B, Kanna RM, et al. Clinical efficacy of ultrasound guided bilateral erector spinae block for single-level lumbar fusion surgery: a prospective, randomized, case-control study. Spine J. 2021;21(11):1873-80.
105. Goldstein CL, Macwan K, Sundararajan K, Rampersaud YR. Perioperative outcomes and adverse events of minimally invasive versus open posterior lumbar fusion: meta-analysis and systematic review. J Neurosurg Spine. 2016;24(3):416-27.

106. Gong J, Luo L, Liu H, Li C, Tang Y, Zhou Y. How Much Benefit Can Patients Acquire from Enhanced Recovery After Surgery Protocols with Percutaneous Endoscopic Lumbar Interbody Fusion? Int J Gen Med. 2021;14:3125-32.
107. Gornitzky AL, Flynn JM, Muhly WT, Sankar WN. A Rapid Recovery Pathway for Adolescent Idiopathic Scoliosis That Improves Pain Control and Reduces Time to Inpatient Recovery After Posterior Spinal Fusion. Spine deformity. 2016;4(4):288-95.
108. Govil N, Parag K, Arora P, Khandelwal H, Singh A. Perioperative duloxetine as part of a multimodal analgesia regime reduces postoperative pain in lumbar canal stenosis surgery: a randomized, triple blind, and placebo-controlled trial. Korean J Pain. 2020;33(1):40-7.
109. Grabel ZJ, Boden A, Segal DN, Boden S, Milby AH, Heller JG. The impact of prophylactic intraoperative vancomycin powder on microbial profile, antibiotic regimen, length of stay, and reoperation rate in elective spine surgery. Spine J. 2019;19(2):261-6.
110. Grasu RM, Cata JP, Dang AQ, Tatsui CE, Rhines LD, Hagan KB, et al. Implementation of an Enhanced Recovery After Spine Surgery program at a large cancer center: a preliminary analysis. J Neurosurg Spine. 2018;29(5):588-98.
111. Grocott MP, Dushianthan A, Hamilton MA, Mythen MG, Harrison D, Rowan K. Perioperative increase in global blood flow to explicit defined goals and outcomes after surgery: a Cochrane Systematic Review. Br J Anaesth. 2013;111(4):535-48.
112. Guan J, Cole CD, Schmidt MH, Dailey AT. Utility of intraoperative rotational thromboelastometry in thoracolumbar deformity surgery. J Neurosurg Spine. 2017;27(5):528-33.
113. Guilfoyle MR, Mannion RJ, Mitchell P, Thomson S. Epidural fentanyl for postoperative analgesia after lumbar canal decompression: a randomized controlled trial. Spine J. 2012;12(8):646-51.
114. Gurbet A, Bekar A, Bilgin H, Korfali G, Yilmazlar S, Tercan M. Pre-emptive infiltration of levobupivacaine is superior to at-closure administration in lumbar laminectomy patients. Eur Spine J. 2008;17(9):1237-41.
115. Hadi BA, Al Ramadani R, Daas R, Naylor I, Zelko R, Saleh M. The influence of anaesthetic drug selection for scoliosis surgery on the management of intraoperative haemodynamic stability and postoperative pain—pharmaceutical care programme. Southern African Journal of Anaesthesia and Analgesia. 2009;15(5):10-4.
116. Hans P, Brichant JF, Bonhomme V, Triffaux M. Analgesic efficiency of propacetamol hydrochlorid after lumbar disc surgery. Acta Anaesthesiol Belg. 1993;44(4):129-33.
117. Hawasli AH, Ray WZ, Goad MA, Frank TL, Ellis ER, Schmidt M, et al. Project management for developing a spine "enhanced recovery after surgery" program in a large university-affiliated hospital. J Neurosurg Sci. 2020;64(2):206-12.
118. He B, Li Y, Xu S, Ou Y, Zhao J. Tranexamic Acid for Blood Loss after Transforaminal Posterior Lumbar Interbody Fusion Surgery: A Double-Blind, Placebo-Controlled, Randomized Study. Biomed Res Int. 2020;2020:8516504.
119. Heathcote S, Sr., Duggan K, Rosbrugh J, Hill B, Shaker R, Hope WW, et al. Enhanced Recovery after Surgery (ERAS) Protocols Expanded over Multiple Service Lines Improves Patient Care and Hospital Cost. Am Surg. 2019;85(9):1044-50.
120. Heo DH, Jang JW, Park CK. Enhanced recovery after surgery pathway with modified biportal endoscopic transforaminal lumbar interbody fusion using a large cage. Comparative study with minimally invasive microscopic transforaminal lumbar interbody fusion. European spine journal : official publication of the European Spine Society, the European Spinal Deformity

Society, and the European Section of the Cervical Spine Research Society. 2023;32(8):2853-62.
121. Heo DH, Park CK. Clinical results of percutaneous biportal endoscopic lumbar interbody fusion with application of enhanced recovery after surgery. Neurosurg Focus. 2019;46(4):E18.
122. Hernández-Palazón J, Tortosa JA, Martínez-Lage JF, Pérez-Flores D. Intravenous administration of propacetamol reduces morphine consumption after spinal fusion surgery. Anesth Analg. 2001;92(6):1473-6.
123. Heroabadi A, Adeli S, Varpaei HA. Accelerating Remobilization Time Following Spine Surgery Using Enhanced Recovery After Surgery: A Randomized Controlled Trial. Archives of Neuroscience 2023 10:2. 2023;10(2):133609-.
124. Hill BW, Emohare O, Song B, Davis R, Kang MM. The use of vancomycin powder reduces surgical reoperation in posterior instrumented and noninstrumented spinal surgery. Acta Neurochir (Wien). 2014;156(4):749-54.
125. Hui S, Xu D, Ren Z, Chen X, Sheng L, Zhuang Q, et al. Can tranexamic acid conserve blood and save operative time in spinal surgeries? A meta-analysis. Spine J. 2018;18(8):1325-37.
126. Hurlbert RJ, Theodore N, Drabier JB, Magwood AM, Sonntag VK. A prospective randomized double-blind controlled trial to evaluate the efficacy of an analgesic epidural paste following lumbar decompressive surgery. J Neurosurg. 1999;90(2 Suppl):191-7.
127. Ibrahim A, Aly M, Farrag W. Effect of intravenous lidocaine infusion on long-term postoperative pain after spinal fusion surgery. Medicine (Baltimore). 2018;97(13):e0229.
128. Ifrach J, Basu R, Joshi DS, Flanders TM, Ozturk AK, Malhotra NR, et al. Efficacy of an Enhanced Recovery After Surgery (ERAS) Pathway in Elderly Patients Undergoing Spine and Peripheral Nerve Surgery. Clin Neurol Neurosurg. 2020;197:106115.
129. Javaherforooshzadeh F, Amirpour I, Janatmakan F, Soltanzadeh M. Comparison of Effects of Melatonin and Gabapentin on Post Operative Anxiety and Pain in Lumbar Spine Surgery: A Randomized Clinical Trial. Anesth Pain Med. 2018;8(3):e68763.
130. Javery KB, Ussery TW, Steger HG, Colclough GW. Comparison of morphine and morphine with ketamine for postoperative analgesia. Can J Anaesth. 1996;43(3):212-5.
131. Jazini E, Thomson AE, Sabet AD, Carreon LY, Roy R, Haines CM, et al. Adoption of Enhanced Surgical Recovery (ESR) Protocol for Lumbar Fusion Decreases In-Hospital Postoperative Opioid Consumption. Global Spine J. 2023;13(4):1030-5.
132. Jian K, Cui J, Li C, Liu R. The Comparison of enhanced recovery after surgery versus traditional pathway in early-onset scoliosis surgery. medRxiv. 2023:2023.04.08.23288328-2023.04.08.
133. Jin Y, Zhao S, Cai J, Blessing M, Zhao X, Tan H, et al. Erector Spinae Plane Block for Perioperative Pain Control and Short-term Outcomes in Lumbar Laminoplasty: A Randomized Clinical Trial. J Pain Res. 2021;14:2717-27.
134. Jirarattanaphochai K, Jung S. Nonsteroidal antiinflammatory drugs for postoperative pain management after lumbar spine surgery: a meta-analysis of randomized controlled trials. J Neurosurg Spine. 2008;9(1):22-31.
135. Jirarattanaphochai K, Jung S, Thienthong S, Krisanaprakornkit W, Sumananont C. Peridural methylprednisolone and wound infiltration with bupivacaine for postoperative pain control after posterior lumbar spine surgery: a randomized double-blinded placebo-controlled trial. Spine (Phila Pa 1976). 2007;32(6):609-16; discussion 17.

136. Jirarattanaphochai K, Thienthong S, Sriraj W, Jung S, Pulnitiporn A, Lertsinudom S, et al. Effect of parecoxib on postoperative pain after lumbar spine surgery: a bicenter, randomized, double-blinded, placebo-controlled trial. Spine (Phila Pa 1976). 2008;33(2):132-9.
137. Julien-Marsollier F, Michelet D, Assaker R, Doval A, Louisy S, Madre C, et al. Enhanced recovery after surgical correction of adolescent idiopathic scoliosis. Paediatr Anaesth. 2020;30(10):1068-76.
138. Kalinin AA, Goloborodko VY, Shepelev VV, Pestryakov YY, Biryuchkov MY, Satardinova EE, et al. Accelerated Recovery Program for Patients with Polysegmental Degenerative Lumbar Spine Disease. Sovrem Tekhnologii Med. 2021;13(2):74-81.
139. Kang H, Jung HJ, Lee JS, Yang JJ, Shin HY, Song KS. Early postoperative analgesic effects of a single epidural injection of ropivacaine administered preoperatively in posterior lumbar interbody spinal arthrodesis: a pilot randomized controlled trial. J Bone Joint Surg Am. 2013;95(5):393-9.
140. Kelly MP, Zebala LP, Kim HJ, Sciubba DM, Smith JS, Shaffrey CI, et al. Effectiveness of preoperative autologous blood donation for protection against allogeneic blood exposure in adult spinal deformity surgeries: a propensity-matched cohort analysis. J Neurosurg Spine. 2016;24(1):124-30.
141. Kerolus MG, Yerneni K, Witiw CD, Shelton A, Canar WJ, Daily D, et al. Enhanced Recovery After Surgery Pathway for Single-Level Minimally Invasive Transforaminal Lumbar Interbody Fusion Decreases Length of Stay and Opioid Consumption. Neurosurgery. 2021;88(3):648-57.
142. Kesänen J, Leino-Kilpi H, Lund T, Montin L, Puukka P, Valkeapää K. The Knowledge Test Feedback Intervention (KTFI) increases knowledge level of spinal stenosis patients before operation-A randomized controlled follow-up trial. Patient Educ Couns. 2016;99(12):1984-91.
143. Kesimci E, Gümüş T, Izdeş S, Sen P, Kanbak O. Comparison of efficacy of dexketoprofen versus paracetamol on postoperative pain and morphine consumption in laminectomy patients. Agri. 2011;23(4):153-9.
144. Khajavi MR, Asadian MA, Imani F, Etezadi F, Moharari RS, Amirjamshidi A. General anesthesia versus combined epidural/general anesthesia for elective lumbar spine disc surgery: A randomized clinical trial comparing the impact of the two methods upon the outcome variables. Surg Neurol Int. 2013;4:105.
145. Khajavikhan J, Vasigh A, Jaafarpour M, Khani A. A multimodal analgesia of cyclooxygenase-2 for postoperative pain. Der pharmacia lettre. 2016;8(8):113-20.
146. Khan ZH, Rahimi M, Makarem J, Khan RH. Optimal dose of pre-incision/post-incision gabapentin for pain relief following lumbar laminectomy: a randomized study. Acta Anaesthesiol Scand. 2011;55(3):306-12.
147. Khanna R, Harris DA, McDevitt JL, Fessler RG, Carabini LM, Lam SK, et al. Impact of Anemia and Transfusion on Readmission and Length of Stay After Spinal Surgery: A Single-center Study of 1187 Operations. Clin Spine Surg. 2017;30(10):E1338-e42.
148. Khurana G, Jindal P, Sharma JP, Bansal KK. Postoperative pain and long-term functional outcome after administration of gabapentin and pregabalin in patients undergoing spinal surgery. Spine (Phila Pa 1976). 2014;39(6):E363-8.
149. Kien NT, Geiger P, Van Chuong H, Cuong NM, Van Dinh N, Pho DC, et al. Preemptive analgesia after lumbar spine surgery by pregabalin and celecoxib: a prospective study. Drug Des Devel Ther. 2019;13:2145-52.

150. Kilic ET, Tastan NO, Sarikaya C, Naderi S. The Application of an Enhanced Recovery After Surgery to Spine Instrumentation. Turk Neurosurg. 2020;30(5):707-13.
151. Kim E, Lee B, Cucchiaro G. Perioperative Surgical Home: Evaluation of a New Protocol Focused on a Multidisciplinary Approach to Manage Children Undergoing Posterior Spinal Fusion Operation. Anesthesia and Analgesia. 2017;125(3):812-9.
152. Kim EJ, Shim JK, Soh S, Song JW, Lee SR, Kwak YL. Patient-controlled Analgesia With Propacetamol-Fentanyl Mixture for Prevention of Postoperative Nausea and Vomiting in High-risk Patients Undergoing Spine Surgery: A Randomized Controlled Trial. J Neurosurg Anesthesiol. 2016;28(4):316-22.
153. Kim HJ, Steinhaus M, Punyala A, Shah S, Elysee JC, Lafage R, et al. Enhanced recovery pathway in adult patients undergoing thoracolumbar deformity surgery. Spine J. 2021;21(5):753-64.
154. Kim KT, Cho DC, Sung JK, Kim YB, Kang H, Song KS, et al. Intraoperative systemic infusion of lidocaine reduces postoperative pain after lumbar surgery: a double-blinded, randomized, placebo-controlled clinical trial. Spine J. 2014;14(8):1559-66.
155. Kim SI, Ha KY, Oh IS. Preemptive multimodal analgesia for postoperative pain management after lumbar fusion surgery: a randomized controlled trial. Eur Spine J. 2016;25(5):1614-9.
156. Kjærgaard M, Møiniche S, Olsen KS. Wound infiltration with local anesthetics for post-operative pain relief in lumbar spine surgery: a systematic review. Acta Anaesthesiol Scand. 2012;56(3):282-90.
157. Klein JD, Hey LA, Yu CS, Klein BB, Coufal FJ, Young EP, et al. Perioperative nutrition and postoperative complications in patients undergoing spinal surgery. Spine (Phila Pa 1976). 1996;21(22):2676-82.
158. Konstantopoulos K, Makris A, Moustaka A, Karmaniolou I, Konstantopoulos G, Mela A. Sevoflurane versus propofol anesthesia in patients undergoing lumbar spondylodesis: A randomized trial. Journal of Surgical Research. 2013;179(1):72-7.
159. Kou L, Wan W, Chen C, Zhao D, Sun X, Gao Z, et al. Can the Full-Percutaneous Endoscopic Lumbar Discectomy in Day Surgery Mode Achieve Better Outcomes Following Enhanced Recovery after Surgery Protocol? A Retrospective Comparative Study. Frontiers in Surgery. 2022;9.
160. Kraiwattanapong C, Arnuntasupakul V, Kantawan R, Woratanarat P, Keorochana G, Langsanam N. Effect of Multimodal Drugs Infiltration on Postoperative Pain in Split Laminectomy of Lumbar Spine: A Randomized Controlled Trial. Spine (Phila Pa 1976). 2020;45(24):1687-95.
161. Kumar G, Stendall C, Mistry R, Gurusamy K, Walker D. A comparison of total intravenous anaesthesia using propofol with sevoflurane or desflurane in ambulatory surgery: systematic review and meta-analysis. Anaesthesia. 2014;69(10):1138-50.
162. Kurnutala LN, Dibble JE, Kinthala S, Tucci MA. Enhanced Recovery After Surgery Protocol for Lumbar Spinal Surgery With Regional Anesthesia: A Retrospective Review. Cureus. 2021;13(9).
163. Lampilas A, Bouyer B, Ferrero E, Khalifé M, Bergeot A, Guigui P, et al. Evaluation of enhanced recovery after spine surgery: Specificities in an academic public hospital. Orthop Traumatol Surg Res. 2021;107(7):103027.

164. Lee BH, Park JO, Suk KS, Kim TH, Lee HM, Park MS, et al. Pre-emptive and multi-modal perioperative pain management may improve quality of life in patients undergoing spinal surgery. Pain Physician. 2013;16(3):E217-26.
165. Lee CH, Liu JT, Lin SC, Hsu TY, Lin CY, Lin LY. Effects of Educational Intervention on State Anxiety and Pain in People Undergoing Spinal Surgery: A Randomized Controlled Trial. Pain Manag Nurs. 2018;19(2):163-71.
166. Lee S, Kim CH, Chung CK, Park SB, Yang SH, Kim SH, et al. Risk factor analysis for postoperative urinary retention after surgery for degenerative lumbar spinal stenosis. Spine J. 2017;17(4):469-77.
167. Leng X, Zhang Y, Wang G, Liu L, Fu J, Yang M, et al. An enhanced recovery after surgery pathway: LOS reduction, rapid discharge and minimal complications after anterior cervical spine surgery. BMC Musculoskelet Disord. 2022;23(1):252.
168. Li J, Li H, Xv ZK, Wang J, Yu QF, Chen G, et al. Enhanced recovery care versus traditional care following laminoplasty: A retrospective case-cohort study. Medicine (Baltimore). 2018;97(48):e13195.
169. Li J, Yang JS, Dong BH, Ye JM. The Effect of Dexmedetomidine Added to Preemptive Ropivacaine Infiltration on Postoperative Pain After Lumbar Fusion Surgery: A Randomized Controlled Trial. Spine (Phila Pa 1976). 2019;44(19):1333-8.
170. Li Q, Zhang Z, Cai Z. High-dose ketorolac affects adult spinal fusion: a meta-analysis of the effect of perioperative nonsteroidal anti-inflammatory drugs on spinal fusion. Spine (Phila Pa 1976). 2011;36(7):E461-8.
171. Li Y, Lu S, Ma SC, Fan HW, Zhao GQ. Effects of Patient-Controlled Epidural Analgesia and Patient-Controlled Intravenous Analgesia on Analgesia in Patients Undergoing Spinal Fusion Surgery. Am J Ther. 2016;23(6):e1806-e12.
172. Li ZE, Lu SB, Kong C, Sun WZ, Wang P, Zhang ST. Impact of Compliance with an Enhanced Recovery After Surgery Program on the Outcomes Among Elderly Patients Undergoing Lumbar Fusion Surgery. Clin Interv Aging. 2020;15:2423-30.
173. Li ZE, Lu SB, Kong C, Sun WZ, Wang P, Zhang ST. Comparative short-term outcomes of enhanced recovery after surgery (ERAS) program and non-ERAS traditional care in elderly patients undergoing lumbar arthrodesis: a retrospective study. BMC Musculoskelet Disord. 2021;22(1):283.
174. Lin WL, Lee MS, Wong CS, Chan SM, Lai HC, Wu ZF, et al. Effects of intraoperative propofol-based total intravenous anesthesia on postoperative pain in spine surgery: Comparison with desflurane anesthesia - a randomised trial. Medicine (Baltimore). 2019;98(13):e15074.
175. Lindbäck Y, Tropp H, Enthoven P, Abbott A, Öberg B. PREPARE: presurgery physiotherapy for patients with degenerative lumbar spine disorder: a randomized controlled trial. Spine J. 2018;18(8):1347-55.
176. Liounakos JI, Wang MY. The Endoscopic Approach to Lumbar Discectomy, Fusion, and Enhanced Recovery: A Review. Global Spine Journal. 2020;10(2_suppl):65S-9S.
177. Liu B, Liu S, Wang Y, Zhao L, Zheng T, Chen L, et al. Enhanced Recovery After Intraspinal Tumor Surgery: A Single-Institutional Randomized Controlled Study. World Neurosurg. 2020;136:e542-e52.
178. Liu D, Zhang G, Zhu Y, Liu X, Xu S, He M, et al. Effectiveness of Ultrasound-Guided Retrolaminar Block and Erector Spinae Plane Block in Retroperitoneal Laparoscopic Surgery: A Randomized Controlled Trial. J Pain Res. 2022;15:815-26.

179. Lloyd TD, Geneen LJ, Bernhardt K, McClune W, Fernquest SJ, Brown T, et al. Cell salvage for minimising perioperative allogeneic blood transfusion in adults undergoing elective surgery. Cochrane Database of Systematic Reviews. 2023(9).
180. Loftus RW, Yeager MP, Clark JA, Brown JR, Abdu WA, Sengupta DK, et al. Intraoperative ketamine reduces perioperative opiate consumption in opiate-dependent patients with chronic back pain undergoing back surgery. Anesthesiology. 2010;113(3):639-46.
181. Lotfinia I, Khallaghi E, Meshkini A, Shakeri M, Shima M, Safaeian A. Interaoperative use of epidural methylprednisolone or bupivacaine for postsurgical lumbar discectomy pain relief: a randomized, placebo-controlled trial. Ann Saudi Med. 2007;27(4):279-83.
182. Lotzke H, Brisby H, Gutke A, Hägg O, Jakobsson M, Smeets R, et al. A Person-Centered Prehabilitation Program Based on Cognitive-Behavioral Physical Therapy for Patients Scheduled for Lumbar Fusion Surgery: A Randomized Controlled Trial. Phys Ther. 2019;99(8):1069-88.
183. Louw A, Diener I, Landers MR, Puentedura EJ. Preoperative pain neuroscience education for lumbar radiculopathy: a multicenter randomized controlled trial with 1-year follow-up. Spine (Phila Pa 1976). 2014;39(18):1449-57.
184. Lu HR, Yang A, Li X, He MZ, Sun JY. A new nursing pattern based on ERAS concept for patients with lumbar degenerative diseases treated with OLIF surgery: A retrospective study. Frontiers in Surgery. 2023;10:1121807-.
185. Luo W, Sun RX, Jiang H, Ma XL. The effect of diabetes on perioperative complications following spinal surgery: a meta-analysis. Ther Clin Risk Manag. 2018;14:2415-23.
186. M R El Ghamry ASE, A G Anwar, M N Shaddad. The Ultrasound-guided erector spinae plane block for acute pain management in patients undergoing posterior lumbar interbody fusion under general anaesthesia. Southern African Journal of Anaesthesia and Analgesia. 2019;Southern African Journal of Anaesthesia and Analgesia 2019; 25(6):26-31.
187. Maheshwari K, Avitsian R, Sessler DI, Makarova N, Tanios M, Raza S, et al. Multimodal Analgesic Regimen for Spine Surgery: A Randomized Placebo-controlled Trial. Anesthesiology. 2020;132(5):992-1002.
188. Marek RJ, Block AR, Ben-Porath YS. The Minnesota Multiphasic Personality Inventory-2-Restructured Form (MMPI-2-RF): incremental validity in predicting early postoperative outcomes in spine surgery candidates. Psychol Assess. 2015;27(1):114-24.
189. Mariappan R, Mehta J, Massicotte E, Nagappa M, Manninen P, Venkatraghavan L. Effect of superficial cervical plexus block on postoperative quality of recovery after anterior cervical discectomy and fusion: a randomized controlled trial. Can J Anaesth. 2015;62(8):883-90.
190. McClelland S, 3rd, Takemoto RC, Lonner BS, Andres TM, Park JJ, Ricart-Hoffiz PA, et al. Analysis of Postoperative Thoracolumbar Spine Infections in a Prospective Randomized Controlled Trial Using the Centers for Disease Control Surgical Site Infection Criteria. Int J Spine Surg. 2016;10:14.
191. McGlew IC, Angliss DB, Gee GJ, Rutherford A, Wood AT. A comparison of rectal indomethacin with placebo for pain relief following spinal surgery. Anaesth Intensive Care. 1991;19(1):40-5.
192. McGuire KJ, Khaleel MA, Rihn JA, Lurie JD, Zhao W, Weinstein JN. The effect of high obesity on outcomes of treatment for lumbar spinal conditions: subgroup analysis of the spine patient outcomes research trial. Spine (Phila Pa 1976). 2014;39(23):1975-80.

193. Michael Y. Wang P-YC. Development of an Enhanced Recovery After Surgery (ERAS) approach for lumbar spinal fusion. J Neurosurg Spine. 2017;26(4):411-8.
194. Milbrandt TA, Singhal M, Minter C, McClung A, Talwalkar VR, Iwinski HJ, et al. A comparison of three methods of pain control for posterior spinal fusions in adolescent idiopathic scoliosis. Spine. 2009;34(14):1499-503.
195. Milligan KR, Macafee AL, Fogarty DJ, Wallace RG, Ramsey P. Intraoperative bupivacaine diminishes pain after lumbar discectomy. A randomised double-blind study. J Bone Joint Surg Br. 1993;75(5):769-71.
196. Mishra L, Pradhan S, Pradhan C. Comparison of propofol based anaesthesia to conventional inhalational general anaesthesia for spine surgery. J Anaesthesiol Clin Pharmacol. 2011;27(1):59-61.
197. Muhly WT, Sankar WN, Ryan K, Norton A, Maxwell LG, DiMaggio T, et al. Rapid recovery pathway after spinal fusion for idiopathic scoliosis. Pediatrics. 2016;137(4).
198. Naik BI, Dunn LK, Wanchek TN. Incremental Cost-effectiveness Analysis on Length of Stay of an Enhanced Recovery After Spine Surgery Program: A Single-center, Retrospective Cohort Study. Journal of neurosurgical anesthesiology. 2023;35(2):187-93.
199. Naik BI, Nemergut EC, Kazemi A, Fernández L, Cederholm SK, McMurry TL, et al. The Effect of Dexmedetomidine on Postoperative Opioid Consumption and Pain After Major Spine Surgery. Anesth Analg. 2016;122(5):1646-53.
200. Nazarenko AG, Konovalov NA, Krut'ko AV, Zamiro TN, Geroeva IB, Gubaydullin RR, et al. Postoperative applications of the fast track technology in patients with herniated intervertebral discs of the lumbosacral spine. Zh Vopr Neirokhir Im N N Burdenko. 2016;80(4):5-12.
201. Ng O, Keeler BD, Mishra A, Simpson JA, Neal K, Al-Hassi HO, et al. Iron therapy for preoperative anaemia. Cochrane Database Syst Rev. 2019;12(12):Cd011588.
202. Nielsen PR, Andreasen J, Asmussen M, Tønnesen H. Costs and quality of life for prehabilitation and early rehabilitation after surgery of the lumbar spine. BMC Health Serv Res. 2008;8:209.
203. Nielsen RV, Siegel H, Fomsgaard JS, Andersen JDH, Martusevicius R, Mathiesen O, et al. Preoperative dexamethasone reduces acute but not sustained pain after lumbar disk surgery: a randomized, blinded, placebo-controlled trial. Pain. 2015;156(12):2538-44.
204. Nikooseresht M, Seifrabiei MA, Davoodi M, Aghajanlou M, Sardari MT. Diclofenac Suppository vs. IV Acetaminophen Combined With IV PCA for Postoperative Pain Management in Patients Undergoing Laminectomy: A Randomized, Double-Blinded Clinical Trial. Anesth Pain Med. 2016;6(3):e36812.
205. Oetgen ME, Martin BD, Gordish-Dressman H, Cronin J, Pestieau SR. Effectiveness and Sustainability of a Standardized Care Pathway Developed with Use of Lean Process Mapping for the Treatment of Patients Undergoing Posterior Spinal Fusion for Adolescent Idiopathic Scoliosis. The Journal of bone and joint surgery American volume. 2018;100(21):1864-70.
206. Oguzhan N, Gunday I, Turan A. Effect of magnesium sulfate infusion on sevoflurane consumption, hemodynamics, and perioperative opioid consumption in lumbar disc surgery. J Opioid Manag. 2008;4(2):105-10.
207. Oh TK, Ryu JH, Sohn HM, Jeon YT. Intraoperative Hypothermia Is Associated with Reduced Acute Kidney Injury After Spine Surgery Under General Anesthesia: A Retrospective Observational Study. J Neurosurg Anesthesiol. 2020;32(1):63-9.

208. Olsen MA, Nepple JJ, Riew KD, Lenke LG, Bridwell KH, Mayfield J, et al. Risk Factors for Surgical Site Infection Following Orthopaedic Spinal Operations. JBJS. 2008;90(1):62-9.
209. Onyekwelu I, Glassman SD, Asher AL, Shaffrey CI, Mummaneni PV, Carreon LY. Impact of obesity on complications and outcomes: a comparison of fusion and nonfusion lumbar spine surgery. J Neurosurg Spine. 2017;26(2):158-62.
210. Ozgencil E, Yalcin S, Tuna H, Yorukoglu D, Kecik Y. Perioperative administration of gabapentin 1,200 mg day-1 and pregabalin 300 mg day-1 for pain following lumbar laminectomy and discectomy: a randomised, double-blinded, placebo-controlled study. Singapore Med J. 2011;52(12):883-9.
211. Pandey CK, Navkar DV, Giri PJ, Raza M, Behari S, Singh RB, et al. Evaluation of the optimal preemptive dose of gabapentin for postoperative pain relief after lumbar diskectomy: a randomized, double-blind, placebo-controlled study. J Neurosurg Anesthesiol. 2005;17(2):65-8.
212. Pandey CK, Sahay S, Gupta D, Ambesh SP, Singh RB, Raza M, et al. Preemptive gabapentin decreases postoperative pain after lumbar discoidectomy. Can J Anaesth. 2004;51(10):986-9.
213. Park HY, Sheppard W, Smith R, Xiao J, Gatto J, Bowen R, et al. The combined administration of vancomycin IV, standard prophylactic antibiotics, and vancomycin powder in spinal instrumentation surgery: does the routine use affect infection rates and bacterial resistance? J Spine Surg. 2018;4(2):173-9.
214. Park SY, An HS, Lee SH, Suh SW, Kim JL, Yoon SJ. A prospective randomized comparative study of postoperative pain control using an epidural catheter in patients undergoing posterior lumbar interbody fusion. Eur Spine J. 2016;25(5):1601-7.
215. Passias PG, Poorman GW, Delsole E, Zhou PL, Horn SR, Jalai CM, et al. Adverse Outcomes and Prediction of Cardiopulmonary Complications in Elective Spine Surgery. Global Spine J. 2018;8(3):218-23.
216. Patrick S, McDowell A, Lee A, Frau A, Martin U, Gardner E, et al. Antisepsis of the skin before spinal surgery with povidone iodine-alcohol followed by chlorhexidine gluconate-alcohol versus povidone iodine-alcohol applied twice for the prevention of contamination of the wound by bacteria: a randomised controlled trial. Bone Joint J. 2017;99-b(10):1354-65.
217. Pendi A, Field R, Farhan SD, Eichler M, Bederman SS. Perioperative Ketamine for Analgesia in Spine Surgery: A Meta-analysis of Randomized Controlled Trials. Spine (Phila Pa 1976). 2018;43(5):E299-e307.
218. Pennington Z, Cottrill E, Lubelski D, Ehresman J, Lehner K, Groves ML, et al. Clinical utility of enhanced recovery after surgery pathways in pediatric spinal deformity surgery: Systematic review of the literature. Journal of Neurosurgery: Pediatrics. 2021;27(2):225-38.
219. Pobereskin LH, Sneyd JR. Does wound irrigation with triamcinolone reduce pain after surgery to the lumbar spine? Br J Anaesth. 2000;84(6):731-4.
220. Pobereskin LH, Sneyd JR. Wound infiltration with bupivacaine after surgery to the cervical spine using a posterior approach. British Journal of Anaesthesia. 2000;84(1):87-8.
221. Polat R, Peker K, Gülöksüz Ç T, Ergil J, Akkaya T. Comparison of the postoperative analgesic effects of paracetamol-codeine phosphate and naproxen sodium-codeine phosphate for lumbar disk surgery. Kaohsiung J Med Sci. 2015;31(9):468-72.
222. Pookarnjanamorakot C, Laohacharoensombat W, Jaovisidha S. The clinical efficacy of piroxicam fast-dissolving dosage form for postoperative pain control after simple lumbar spine surgery: a double-blinded randomized study. Spine (Phila Pa 1976). 2002;27(5):447-51.

223. Porche K, Samra R, Melnick K, Brennan M, Vaziri S, Seubert C, et al. Enhanced recovery after surgery (ERAS) for open transforaminal lumbar interbody fusion: a retrospective propensity-matched cohort study. Spine J. 2022;22(3):399-410.
224. Porche K, Yan S, Mohamed B, Garvan C, Samra R, Melnick K, et al. Enhanced recovery after surgery (ERAS) improves return of physiological function in frail patients undergoing one- to two-level TLIFs: an observational retrospective cohort study. The spine journal : official journal of the North American Spine Society. 2022;22(9):1513-22.
225. Porche K, Yan SC, Mehkri Y, Sriram S, MacNeil A, Melnick K, et al. The Enhanced Recovery After Surgery pathway for posterior cervical surgery: a retrospective propensity-matched cohort study. Journal of neurosurgery Spine. 2023;39(2):216-27.
226. Powell R, Scott NW, Manyande A, Bruce J, Vögele C, Byrne-Davis LM, et al. Psychological preparation and postoperative outcomes for adults undergoing surgery under general anaesthesia. Cochrane Database Syst Rev. 2016;2016(5):Cd008646.
227. Prasartritha T, Kunakornsawat S, Tungsiripat R, Jampa J, Throngnumchai R. A prospective randomized trial comparing epidural morphine through intraoperatively placed epidural catheter and intravenous morphine in major lumbar spinal surgery. Journal of spinal disorders & techniques. 2010;23(8).
228. Pull ter Gunne AF, Cohen DB. Incidence, prevalence, and analysis of risk factors for surgical site infection following adult spinal surgery. Spine (Phila Pa 1976). 2009;34(13):1422-8.
229. Raja SD, Shetty AP, Subramanian B, Kanna RM, Rajasekaran S. A prospective randomized study to analyze the efficacy of balanced pre-emptive analgesia in spine surgery. Spine J. 2019;19(4):569-77.
230. Rajpal S, Gordon DB, Pellino TA, Strayer AL, Brost D, Trost GR, et al. Comparison of perioperative oral multimodal analgesia versus IV PCA for spine surgery. Journal of spinal disorders & techniques. 2010;23(2):139-45.
231. Rao KE, Krodel D, Toaz EE, Fanelli J, Hajduk J, Kato K, et al. Introduction of an enhanced recovery pathway results in decreased length of stay in patients with adolescent idiopathic scoliosis undergoing posterior spinal fusion: A description of implementation strategies and retrospective before-and-after study of outcomes. J Clin Anesth. 2021;75:110493.
232. Rao RR, Hayes M, Lewis C, Hensinger RN, Farley FA, Li Y, et al. Mapping the Road to Recovery: Shorter Stays and Satisfied Patients in Posterior Spinal Fusion. Journal of Pediatric Orthopaedics. 2017;37(8):e536-e42.
233. Ren Y, Yu Q-F, Feng X-Q, Ma Y-J, Shen S-Y, Xiao Y-X, et al. Application of enhanced recovery after surgery program for posterior lumbar decompression and fusion. TMR Integrative Nursing. 2019;3(1):35-41.
234. Reuben SS, Connelly NR, Lurie S, Klatt M, Gibson CS. Dose-response of ketorolac as an adjunct to patient-controlled analgesia morphine in patients after spinal fusion surgery. Anesth Analg. 1998;87(1):98-102.
235. Rolving N, Nielsen CV, Christensen FB, Holm R, Bünger CE, Oestergaard LG. Preoperative cognitive-behavioural intervention improves in-hospital mobilisation and analgesic use for lumbar spinal fusion patients. BMC Musculoskelet Disord. 2016;17:217.
236. Salvetti DJ, Tempel ZJ, Gandhoke GS, Parry PV, Grandhi RM, Kanter AS, et al. Preoperative prealbumin level as a risk factor for surgical site infection following elective spine surgery. Surg Neurol Int. 2015;6(Suppl 19):S500-3.

237. Samoladas E, Kapinas A, Papadopoulos DV, Gkiatas I, Papastefanou S, Gelalis ID. Intraoperative epidural application of steroid and local anaesthetic agent following lumbar discectomy: A prospective double blinded randomized controlled trial. J Clin Orthop Trauma. 2019;10(Suppl 1):S143-s6.
238. Sanders AE, Andras LM, Sousa T, Kissinger C, Cucchiaro G, Skaggs DL. Accelerated Discharge Protocol for Posterior Spinal Fusion Patients With Adolescent Idiopathic Scoliosis Decreases Hospital Postoperative Charges 22. Spine. 2017;42(2):92-7.
239. Sanders AE, Andras LM, Sousa T, Kissinger C, Cucchiaro G, Skaggs DL. Accelerated discharge protocol for posterior spinal fusion patients with adolescent idiopathic scoliosis decreases hospital postoperative charges 22%. Spine. 2017;42(2):92-7.
240. Savage JW, Weatherford BM, Sugrue PA, Nolden MT, Liu JC, Song JK, et al. Efficacy of surgical preparation solutions in lumbar spine surgery. J Bone Joint Surg Am. 2012;94(6):490-4.
241. Scanlon J, Richards B. Development of a same day laminectomy program. J Perianesth Nurs. 2004;19(2):84-8.
242. Schenk MR, Putzier M, Kügler B, Tohtz S, Voigt K, Schink T, et al. Postoperative analgesia after major spine surgery: patient-controlled epidural analgesia versus patient-controlled intravenous analgesia. Anesth Analg. 2006;103(5):1311-7.
243. Schraag S, Pradelli L, Alsaleh AJO, Bellone M, Ghetti G, Chung TL, et al. Propofol vs. inhalational agents to maintain general anaesthesia in ambulatory and in-patient surgery: a systematic review and meta-analysis. BMC Anesthesiol. 2018;18(1):162.
244. Sekar C, Rajasekaran S, Kannan R, Reddy S, Shetty TA, Pithwa YK. Preemptive analgesia for postoperative pain relief in lumbosacral spine surgeries: a randomized controlled trial. Spine J. 2004;4(3):261-4.
245. Seng C, Siddiqui MA, Wong KP, Zhang K, Yeo W, Tan SB, et al. Five-year outcomes of minimally invasive versus open transforaminal lumbar interbody fusion: a matched-pair comparison study. Spine (Phila Pa 1976). 2013;38(23):2049-55.
246. Senker W, Gruber A, Gmeiner M, Stefanits H, Sander K, Rössler P, et al. Surgical and Clinical Results of Minimally Invasive Spinal Fusion Surgery in an Unselected Patient Cohort of a Spinal Care Unit. Orthop Surg. 2018;10(3):192-7.
247. Servicl-Kuchler D, Maldini B, Borgeat A, Bilić N, Kosak R, Mavcic B, et al. The influence of postoperative epidural analgesia on postoperative pain and stress response after major spine surgery--a randomized controlled double blind study. Acta Clin Croat. 2014;53(2):176-83.
248. Shao X, Li R, Zhang L, Jiang W. Enhanced Recovery After Surgery Protocol for Oblique Lumbar Interbody Fusion. Indian journal of orthopaedics. 2022;56(6):1073-82.
249. Shaw KA, Fletcher ND, Devito DP, Schmitz ML, Fabregas J, Gidwani S, et al. In-hospital opioid usage following posterior spinal fusion for adolescent idiopathic scoliosis: Does methadone offer an advantage when used with an ERAS pathway? Spine Deform. 2021;9(4):1021-7.
250. Shimia M, Parish M, Abedini N. The effect of intravenous paracetamol on postoperative pain after lumbar discectomy. Asian Spine J. 2014;8(4):400-4.
251. Siam EM, Abo Aliaa DM, Elmedany S, Abdelaa ME. Erector spinae plane block combined with general anaesthesia versus conventional general anaesthesia in lumbar spine surgery. Egyptian Journal of Anaesthesia. 2020;36(1):201-26.

252. Singh K, Bohl DD, Ahn J, Massel DH, Mayo BC, Narain AS, et al. Multimodal Analgesia Versus Intravenous Patient-Controlled Analgesia for Minimally Invasive Transforaminal Lumbar Interbody Fusion Procedures. Spine. 2017;42(15):1145-50.
253. Singh S, Choudhary NK, Lalin D, Verma VK. Bilateral Ultrasound-guided Erector Spinae Plane Block for Postoperative Analgesia in Lumbar Spine Surgery: A Randomized Control Trial. J Neurosurg Anesthesiol. 2020;32(4):330-4.
254. Singhatanadgige W, Chancharoenchai T, Honsawek S, Kotheeranurak V, Tanavalee C, Limthongkul W. No Difference in Pain After Spine Surgery with Local Wound Filtration of Morphine and Ketorolac: A Randomized Controlled Trial. Clin Orthop Relat Res. 2020;478(12):2823-9.
255. Sivaganesan A, Wick JB, Chotai S, Cherkesky C, Stephens BF, Devin CJ. Perioperative Protocol for Elective Spine Surgery Is Associated With Reduced Length of Stay and Complications. The Journal of the American Academy of Orthopaedic Surgeons. 2019;27(5):183-9.
256. Smith J, Probst S, Calandra C, Davis R, Sugimoto K, Nie L, et al. Enhanced recovery after surgery (ERAS) program for lumbar spine fusion. Perioper Med (Lond). 2019;8:4.
257. Soffin EM, Beckman JD, Tseng A, Zhong H, Huang RC, Urban M, et al. Enhanced Recovery after Lumbar Spine Fusion: A Randomized Controlled Trial to Assess the Quality of Patient Recovery. Anesthesiology. 2020;133(2):350-63.
258. Soffin EM, Vaishnav AS, Wetmore DS, Barber L, Hill P, Gang CH, et al. Design and Implementation of an Enhanced Recovery After Surgery (ERAS) Program for Minimally Invasive Lumbar Decompression Spine Surgery: Initial Experience. Spine (Phila Pa 1976). 2019;44(9):E561-e70.
259. Soffin EM, Wetmore DS, Barber LA, Vaishnav AS, Beckman JD, Albert TJ, et al. An enhanced recovery after surgery pathway: association with rapid discharge and minimal complications after anterior cervical spine surgery. Neurosurg Focus. 2019;46(4):E9.
260. Soffin EM, Wetmore DS, Beckman JD, Sheha ED, Vaishnav AS, Albert TJ, et al. Opioid-free anesthesia within an enhanced recovery after surgery pathway for minimally invasive lumbar spine surgery: a retrospective matched cohort study. Neurosurg Focus. 2019;46(4):E8.
261. Solves P, Carpio N, Moscardo F, Bas T, Cañigral C, Salazar C, et al. Results of a preoperative autologous blood donation program for patients undergoing elective major spine surgery. Transfusion and Apheresis Science. 2013;49(2):345-8.
262. Song JW, Shim JK, Song Y, Yang SY, Park SJ, Kwak YL. Effect of ketamine as an adjunct to intravenous patient-controlled analgesia, in patients at high risk of postoperative nausea and vomiting undergoing lumbar spinal surgery. Br J Anaesth. 2013;111(4):630-5.
263. Spreng UJ, Dahl V, Raeder J. Effect of a single dose of pregabalin on post-operative pain and pre-operative anxiety in patients undergoing discectomy. Acta Anaesthesiol Scand. 2011;55(5):571-6.
264. Staartjes VE, de Wispelaere MP, Schröder ML. Improving recovery after elective degenerative spine surgery: 5-year experience with an enhanced recovery after surgery (ERAS) protocol. Neurosurg Focus. 2019;46(4):E7.
265. Steel T, Jones R, Crossman J, Sheehy J, Bentivoglio P, Pell M. Intraoperative wound infiltration with bupivacaine in patients undergoing lumbar spine surgery. J Clin Neurosci. 1998;5(3):298-303.

266. Subramaniam K, Akhouri V, Glazer PA, Rachlin J, Kunze L, Cronin M, et al. Intra- and postoperative very low dose intravenous ketamine infusion does not increase pain relief after major spine surgery in patients with preoperative narcotic analgesic intake. Pain Med. 2011;12(8):1276-83.
267. Sun Z, Qi Y. Application of enhanced recovery after surgery care protocol in the perioperative care of patients undergoing lumbar fusion and internal fixation. J Orthop Surg Res. 2022;17(1):240.
268. Tang J, Fan J, Yao Y, Cai W, Yin G, Zhou W. Application of a buprenorphine transdermal patch for the perioperative analgesia in patients who underwent simple lumbar discectomy. Medicine (Baltimore). 2017;96(20):e6844.
269. Tarıkçı Kılıç E, Demirbilek T, Naderi S. Does an enhanced recovery after surgery protocol change costs and outcomes of single-level lumbar microdiscectomy? Neurosurg Focus. 2019;46(4):E10.
270. Taşkaldıran Y. Is Opioid-free Anesthesia Possible by Using Erector Spinae Plane Block in Spinal Surgery? Cureus. 2021;13(10):e18666.
271. Thomson K, Pestieau SR, Patel JJ, Gordish-Dressman H, Mirzada A, Kain ZN, et al. Perioperative Surgical Home in Pediatric Settings: Preliminary Results. Anesthesia and Analgesia. 2016;123(5):1193-200.
272. Tran S, Wolever TM, Errett LE, Ahn H, Mazer CD, Keith M. Preoperative carbohydrate loading in patients undergoing coronary artery bypass or spinal surgery. Anesth Analg. 2013;117(2):305-13.
273. Trief PM, Ploutz-Snyder R, Fredrickson BE. Emotional health predicts pain and function after fusion: a prospective multicenter study. Spine (Phila Pa 1976). 2006;31(7):823-30.
274. Tripi PA, Poe-Kochert C, Potzman J, Son-Hing JP, Thompson GH. Intrathecal morphine for postoperative analgesia in patients with idiopathic scoliosis undergoing posterior spinal fusion. Spine. 2008;33(20):2248-51.
275. Tsaousi G, Nikopoulou A, Pezikoglou I, Birba V, Grosomanidis V. Implementation of magnesium sulphate as an adjunct to multimodal analgesic approach for perioperative pain control in lumbar laminectomy surgery: A randomized placebo-controlled clinical trial. Clin Neurol Neurosurg. 2020;197:106091.
276. Ueshima H, Hara E, Otake H. RETRACTED: Thoracolumbar interfascial plane block provides effective perioperative pain relief for patients undergoing lumbar spinal surgery; a prospective, randomized and double blinded trial. J Clin Anesth. 2019;58:12-7.
277. Urban MK, Jules-Elysee K, Urquhart B, Cammisa FP, Boachie-Adjei O. Reduction in postoperative pain after spinal fusion with instrumentation using intrathecal morphine. Spine. 2002;27(5):535-7.
278. Urban MK, Ya Deau JT, Wukovits B, Lipnitsky JY. Ketamine as an adjunct to postoperative pain management in opioid tolerant patients after spinal fusions: a prospective randomized trial. Hss j. 2008;4(1):62-5.
279. van Rhee H, Suurmond R. Meta-Analyze Dichotomous Data: Do the Calculations with Log Odds Ratios and Report Risk Ratios or Risk Differences. SSRN Electronic Journal. 2015.
280. Vasigh A, Jaafarpour M, Khajavikhan J, Khani A. The Effect of Gabapentin Plus Celecoxib on Pain and Associated Complications After Laminectomy. J Clin Diagn Res. 2016;10(3):Uc04-8.

281. Vasigh A, Najafi F, Khajavikhan J, Jaafarpour M, Khani A. Comparing Gabapentin and Celecoxib in Pain Management and Complications After Laminectomy: A Randomized Double-Blind Clinical Trial. Iran Red Crescent Med J. 2016;18(2):e34559.
282. Venkata HK, van Dellen JR. A perspective on the use of an enhanced recovery program in open, non-instrumented day surgery for degenerative lumbar and cervical spinal conditions. J Neurosurg Sci. 2018;62(3):245-54.
283. Wainwright TW, Immins T, Middleton RG. Enhanced recovery after surgery (ERAS) and its applicability for major spine surgery. Best Pract Res Clin Anaesthesiol. 2016;30(1):91-102.
284. Wang MY, Chang HK, Grossman J. Reduced Acute Care Costs With the ERAS® Minimally Invasive Transforaminal Lumbar Interbody Fusion Compared With Conventional Minimally Invasive Transforaminal Lumbar Interbody Fusion. Neurosurgery. 2018;83(4):827-34.
285. Wang MY, Grossman J. Endoscopic minimally invasive transforaminal interbody fusion without general anesthesia: initial clinical experience with 1-year follow-up. Neurosurg Focus. 2016;40(2):E13.
286. Wang P, Wang Q, Kong C, Teng Z, Li Z, Zhang S, et al. Enhanced recovery after surgery (ERAS) program for elderly patients with short-level lumbar fusion. J Orthop Surg Res. 2020;15(1):299.
287. Wang S, Wang P, Li X, Sun W, Kong C, Lu S. Enhanced recovery after surgery pathway: association with lower incidence of wound complications and severe hypoalbuminemia in patients undergoing posterior lumbar fusion surgery. Journal of Orthopaedic Surgery and Research. 2022;17(1):178-.
288. Wang W, Wang P, Kong C, Teng Z, Zhang S, Sun W, et al. Retrospective Data Analysis for Enhanced Recovery After Surgery (ERAS) Protocol for Elderly Patients with Long-Level Lumbar Fusion. World Neurosurg. 2022;164:e397-e403.
289. Wang W LY, Zhang Y. Ultrasound-guided erector spine block as an adjuvant to general anesthesia and postoperative analgesia in patients undergoing lumbar spine surgery. Journal of Clinical Anesthesiology. 2018(12).
290. Wang Y, Guo X, Guo Z, Xu M. Preemptive analgesia with a single low dose of intrathecal morphine in multilevel posterior lumbar interbody fusion surgery: a double-blind, randomized, controlled trial. Spine J. 2020;20(7):989-97.
291. Wijeysundera DN, Pearse RM, Shulman MA, Abbott TEF, Torres E, Ambosta A, et al. Assessment of functional capacity before major non-cardiac surgery: an international, prospective cohort study. Lancet. 2018;391(10140):2631-40.
292. Wittayapairoj A, Wittayapairoj K, Kulawong A, Huntula Y. Effect of intermediate dose dexamethasone on post-operative pain in lumbar spine surgery: A randomized, triple-blind, placebo-controlled trial. Asian J Anesthesiol. 2017;55(3):73-7.
293. Wongkietkachorn A, Wongkietkachorn N, Rhunsiri P. Preoperative Needs-Based Education to Reduce Anxiety, Increase Satisfaction, and Decrease Time Spent in Day Surgery: A Randomized Controlled Trial. World J Surg. 2018;42(3):666-74.
294. Wu FY. Influence of rehabilitation education on behavior and lumbar functional exercise in patients following operation for lumbar spinal stenosis. 2005;9:20-2.
295. Wu X XF, Wang J, Yang L, Chen X, Yao S. Analgesic efficacy of ultrasound-guided bilateral erector spinae plane block in patients undergoing posterior lumbar interbody fusion. J Clin Anesthesiology 2019;2019;35(9):842–845.
296. X YQG. Ropivacaine erector spinae plane block assisting general anesthesia in lumbar spine surgery of 30 cases. Herbal Medicine. 2018;Her Med 2018; 37: 63-6. Chinese.

297. Xu B, Ren L, Tu W, Wu Z, Ai F, Zhou D, et al. Continuous wound infusion of ropivacaine for the control of pain after thoracolumbar spinal surgery: a randomized clinical trial. Eur Spine J. 2017;26(3):825-31.
298. Yamauchi M, Asano M, Watanabe M, Iwasaki S, Furuse S, Namiki A. Continuous low-dose ketamine improves the analgesic effects of fentanyl patient-controlled analgesia after cervical spine surgery. Anesth Analg. 2008;107(3):1041-4.
299. Yang J, Skaggs DL, Chan P, Villamor GA, Choi PD, Tolo VT, et al. High Satisfaction in Adolescent Idiopathic Scoliosis Patients on Enhanced Discharge Pathway. J Pediatr Orthop. 2020;40(3):e166-e70.
300. Yang Y, Wu X, Wu W, Liu Z, Pang M, Chen Y, et al. Enhanced recovery after surgery (ERAS) pathway for microendoscopy-assisted minimally invasive transforaminal lumbar interbody fusion. Clin Neurol Neurosurg. 2020;196:106003.
301. Yang YJ, Huang X, Gao XN, Xia B, Gao JB, Wang C, et al. An Optimized Enhanced Recovery After Surgery (ERAS) Pathway Improved Patient Care in Adolescent Idiopathic Scoliosis Surgery: A Retrospective Cohort Study. World Neurosurg. 2021;145:e224-e32.
302. Yayik AM, Cesur S, Ozturk F, Ahiskalioglu A, Ay AN, Celik EC, et al. Postoperative Analgesic Efficacy of the Ultrasound-Guided Erector Spinae Plane Block in Patients Undergoing Lumbar Spinal Decompression Surgery: A Randomized Controlled Study. World Neurosurg. 2019;126:e779-e85.
303. Yeom JH, Chon MS, Jeon WJ, Shim JH. Peri-operative ketamine with the ambulatory elastometric infusion pump as an adjuvant to manage acute postoperative pain after spinal fusion in adults: a prospective randomized trial. Korean J Anesthesiol. 2012;63(1):54-8.
304. Yeom JH, Kim KH, Chon MS, Byun J, Cho SY. Remifentanil used as adjuvant in general anesthesia for spinal fusion does not exhibit acute opioid tolerance. Korean J Anesthesiol. 2012;63(2):103-7.
305. Yeşiltaş S, Abdallah A, Uysal Ö, Yilmaz S, Çinar İ, Karaaslan K. The Efficacy of Intraoperative Freehand Erector Spinae Plane Block in Lumbar Spondylolisthesis: A Randomized Controlled Study. Spine. 2021;46(17):E902-E10.
306. Yörükoğlu D, Ateş Y, Temiz H, Yamali H, Kecik Y. Comparison of low-dose intrathecal and epidural morphine and bupivacaine infiltration for postoperative pain control after surgery for lumbar disc disease. J Neurosurg Anesthesiol. 2005;17(3):129-33.
307. Yörükoğlu HU, İçli D, Aksu C, Cesur S, Kuş A, Gürkan Y. Erector spinae block for postoperative pain management in lumbar disc hernia repair. Journal of Anesthesia. 2021;35(3):420-5.
308. Young R, Cottrill E, Pennington Z, Ehresman J, Ahmed AK, Kim T, et al. Experience with an Enhanced Recovery After Spine Surgery protocol at an academic community hospital. J Neurosurg Spine. 2021;34(4):680-7.
309. Yu Y, Wang M, Ying H, Ding J, Wang H, Wang Y. The Analgesic Efficacy of Erector Spinae Plane Blocks in Patients Undergoing Posterior Lumbar Spinal Surgery for Lumbar Fracture. World Neurosurg. 2021;147:e1-e7.
310. Yuan Y, Wang SK, Chai XY, Wang P, Li XY, Kong C, et al. The implementation of enhanced recovery after surgery pathway in patients undergoing posterior thoracolumbar fusion for degenerative spinal deformity. BMC musculoskeletal disorders. 2023;24(1).
311. Yukawa Y, Kato F, Ito K, Terashima T, Horie Y. A prospective randomized study of preemptive analgesia for postoperative pain in the patients undergoing posterior lumbar

interbody fusion: continuous subcutaneous morphine, continuous epidural morphine, and diclofenac sodium. Spine (Phila Pa 1976). 2005;30(21):2357-61.
312. Zarei M, Najafi A, Mansouri P, Sadeghi-Yazdankhah S, Saberi H, Moradi M, et al. Management of postoperative pain after Lumbar surgery-pregabalin for one day and 14 days-a randomized, triple-blinded, placebo-controlled study. Clin Neurol Neurosurg. 2016;151:37-42.
313. Zhang CH, Yan BS, Xu BS, Ma XL, Yang Q, Liu Y, et al. [Study on feasibility of enhanced recovery after surgery combined with mobile microendoscopic discectomy-transforaminal lumbar interbody fusion in the treatment of lumbar spondylolisthesis]. Zhonghua Yi Xue Za Zhi. 2017;97(23):1790-5.
314. Zhang H, Wang Z, Li K. Clinical application of enhanced recovery after surgery in lumbar disk herniation patients undergoing dynamic stabilization and discectomy. J Back Musculoskelet Rehabil. 2022;35(1):47-53.
315. Zhang JJ, Zhang TJ, Qu ZY, Qiu Y, Hua Z. Erector spinae plane block at lower thoracic level for analgesia in lumbar spine surgery: A randomized controlled trial. World J Clin Cases. 2021;9(19):5126-34.
316. Zhang Q, Wu Y, Ren F, Zhang X, Feng Y. Bilateral ultrasound-guided erector spinae plane block in patients undergoing lumbar spinal fusion: A randomized controlled trial. J Clin Anesth. 2021;68:110090.
317. Zhang Z, Xu H, Zhang Y, Li W, Yang Y, Han T, et al. Nonsteroidal anti-inflammatory drugs for postoperative pain control after lumbar spine surgery: A meta-analysis of randomized controlled trials. J Clin Anesth. 2017;43:84-9.
318. Zhu L, Wang M, Wang X, Wang Y, Chen L, Li J. Changes of Opioid Consumption After Lumbar Fusion Using Ultrasound-Guided Lumbar Erector Spinae Plane Block: A Randomized Controlled Trial. Pain Physician. 2021;24(2):E161-e8.
319. Zuo X, Wang L, He L, Li P, Zhou D, Yang Y. Enhanced Recovery after Surgery Protocol Accelerates Recovery of Lumbar Disc Herniation among Elderly Patients Undergoing Discectomy via Promoting Gastrointestinal Function. Pain research & management. 2021;2021.
